# Yeast Creates a Niche for Symbiotic Lactic Acid Bacteria through Nitrogen Overflow

**DOI:** 10.1016/j.cels.2017.09.002

**Published:** 2017-10-25

**Authors:** Olga Ponomarova, Natalia Gabrielli, Daniel C. Sévin, Michael Mülleder, Katharina Zirngibl, Katsiaryna Bulyha, Sergej Andrejev, Eleni Kafkia, Athanasios Typas, Uwe Sauer, Markus Ralser, Kiran Raosaheb Patil

**Affiliations:** 1 European Molecular Biology Laboratory, Heidelberg 69117, Germany; 2 Institute of Molecular Systems Biology, ETH-Zürich, Zürich 8093, Switzerland; 3 Department of Biochemistry, University of Cambridge, The Francis Crick Institute, London, NW1 1AT, UK

**Keywords:** microbial communities, cross-feeding, metabolic interactions, TORC1, mutualism, metabolomics

## Abstract

Many microorganisms live in communities and depend on metabolites secreted by fellow community members for survival. Yet our knowledge of interspecies metabolic dependencies is limited to few communities with small number of exchanged metabolites, and even less is known about cellular regulation facilitating metabolic exchange. Here we show how yeast enables growth of lactic acid bacteria through endogenous, multi-component, cross-feeding in a readily established community. In nitrogen-rich environments, *Saccharomyces cerevisiae* adjusts its metabolism by secreting a pool of metabolites, especially amino acids, and thereby enables survival of *Lactobacillus plantarum* and *Lactococcus lactis*. Quantity of the available nitrogen sources and the status of nitrogen catabolite repression pathways jointly modulate this niche creation. We demonstrate how nitrogen overflow by yeast benefits *L. plantarum* in grape juice, and contributes to emergence of mutualism with *L. lactis* in a medium with lactose. Our results illustrate how metabolic decisions of an individual species can benefit others.

## Highlights

• Yeast overflows amino acids that enable survival of lactic acid bacteria (LAB)
• Overflow is in proportion to nitrogen excess and regulated via TORC1 pathway
• Phenotype supporting LAB growth is conserved across diverse yeast isolates
• Yeast-LAB mutualism readily emerges when lactose is the main C-source

## Graphical Abstract

**Figure:**
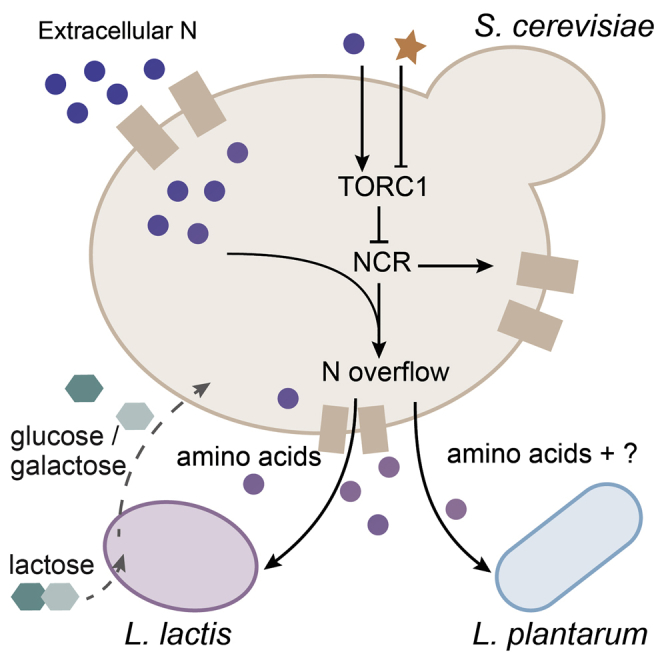

## Teaser

Yeast and LAB co-occur in a variety of naturally fermented foods and beverages. We show, using metabolomics, transcriptomics, and genetic analysis, how nitrogen overflow by yeast benefits LAB and contributes to the emergence of mutualism.

## Introduction

One of the most impactful ecological roles of interspecies metabolite exchange is nutrient cross-feeding. Trophic interactions (food chains) enable multiple groups of organisms to survive on limited resources, increase community diversity, and, on a global scale, drive elemental cycles. For example, in methanogenic anaerobic consortia methane producers rely on electron carriers (hydrogen, acetate, formate, and CO_2_) supplied by primary fermenters (Embree et al., 2015, Morris et al., 2013, Stams, 1994, Tveit et al., 2015); human gut symbionts degrade complex dietary polysaccharides sequentially, by sharing intermediate metabolites with other community members (Koropatkin et al., 2012, Rakoff-Nahoum et al., 2014, Rios-Covian et al., 2015); and soil bacteria secrete diverse array of metabolites that are available for the other community members (Baran et al., 2015). As illustrated in a recent study (Hom and Murray, 2014), symbiotic metabolic interactions such as these can be readily established through compatibility of nutritional requirements and biosynthetic capabilities of different species. Survival of individual species is thus often contingent on the niche created by metabolic activities of the others.

To circumvent challenges of detecting metabolite exchange in complex natural environments, details of metabolic interactions are usually scrutinized in synthetic communities. These are often constructed using known, evolved, or genetically engineered metabolic interactions (Andrade-Dominguez et al., 2014, Hom and Murray, 2014, Kosina et al., 2016, Mee et al., 2014, Miller et al., 2010, Wintermute and Silver, 2010, Zhou et al., 2015), and have provided insights into principles and mechanics of interspecies dependencies (De Roy et al., 2014, Ponomarova and Patil, 2015, Song et al., 2014). Yet, due to selection or engineering, cross-feeding in such communities is limited to few metabolites, and thus may not represent the complexity of naturally occurring interactions. Furthermore, little is known about the regulatory decisions that prompt a microorganism to secrete valuable metabolites that form the basis of interspecies metabolite exchange.

Here we report *de novo* assembly of a stable community of yeast and commensalistic lactic acid bacteria (LAB) with endogenous metabolic dependencies. We use this community to identify novel metabolic exchanges, to demonstrate transition from a unilateral to mutualistic cross-feeding and, perhaps most importantly, to capture the innate complexity of natural interactions and their regulatory underpinnings.

## Results

### Yeast Enables Growth of LAB in a Stable Three-Species Community

As a model system to detect and study metabolic interactions, we set out to compose a three-species community using wild-type *Saccharomyces cerevisiae*, *Lactococcus lactis*, and *Lactobacillus plantarum*. The choice of these species was inspired by recurrent symbiosis of yeast and LAB in a variety of naturally fermented foods and beverages, including kefir, kimchi, wine, sourdough, and cocoa (Blandino et al., 2003, Di Cagno et al., 2014, Lee et al., 2015, Lonvaud-Funel, 2015, Papalexandratou and Nielsen, 2016, Prado et al., 2015, Tamang et al., 2016). Moreover, metabolic interactions between yeast and LAB in these environments have been previously suggested (Gobbetti et al., 1995) and observed for several species (Mendes et al., 2013, Narvhus, 2003, Stadie et al., 2013).

**Figure 1:**
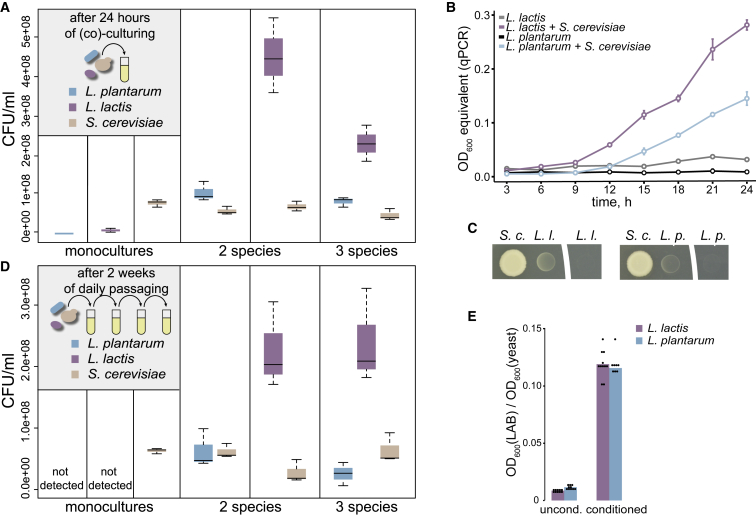
*S. cerevisiae* Enables Growth of Lactic Acid Bacteria (A) Quantification of *S. cerevisiae*, *L. lactis*, and *L. plantarum* colony-forming units (CFUs) in monocultures and communities after 24 hr (pooled technical replicates of n = 3 biological replicates). (B) Dynamics of *L. plantarum* and *L. lactis* growth in co-culture with *S. cerevisiae* (colored lines) and in monoculture (gray lines). Data shown as mean ± SD for three biological replicates. (C) Growth of *L. lactis* (*L.l.*) and *L. plantarum* (*L.p.*) in close proximity and apart from an *S. cerevisiae* (*S.c.*) colony. (D) Quantification of *S. cerevisiae*, *L. lactis*, and *L. plantarum* CFUs in monocultures and communities after 2 weeks of daily passaging (pooled technical replicates of n = 3 biological replicates). See also Figure S1. (E) Effect of yeast-conditioned medium on LAB, normalized by yeast cell density. Barplot shows the mean and the dots represent pooled technical replicates from at least four independent experiments.

To probe for potential metabolic dependencies between selected yeast and LAB species, we first checked whether any of the three microorganisms would show improved growth in co-cultures. We assessed the growth of all three species in mono- and co-cultures in a number of chemically defined media with varying nutritional richness. While yeast can grow in a relatively simple medium, LAB species are fastidious and require many more nutrients, e.g., amino acids and vitamins. A medium for detecting growth-promoting metabolite exchange should strike a balance between scarcity and richness: it should lack components (and their metabolic equivalents) that could be exchanged between community members, but also be sufficiently rich to support the growth of all three species. We started with a rich medium including all requirements of the three species, then prepared variations of this medium by removing single or groups of components. Resulting media were tested to select those supporting growth of microorganisms in co-cultures, but not in monocultures, which indicated nutrient cross-feeding. Among the tested media, one with 35 components (CDM35; Table S1, including eight amino acids, arginine, asparagine, histidine, isoleucine, leucine, methionine, valine, and tyrosine, and ammonium as nitrogen sources) fulfilled this requirement, revealing yeast interaction with both LAB species. While yeast growth varied very little between monoculture and co-culture with LAB in CDM35, both *L. lactis* and *L. plantarum* could grow only when co-cultured with yeast, in both liquid and solid media, suggesting metabolic dependency (Figures 1A–1C).

We tested the stability of this positive interaction by extended co-culturing of yeast and LAB for over 2 weeks with daily passaging into fresh medium. Colony-forming unit-based quantification at the end of the passaging showed that both LAB continue to survive in the presence of yeast, with *L. lactis* being the most abundant member of the community (Figure 1D). Notably, none of the LAB could be detected in passaged monocultures and could survive only in co-cultures with yeast. This stability of the yeast-LAB community was also evident after daily transfers for over 50 days as well as in a continuous weeklong co-culture (Figure S1).

### Yeast-LAB Interaction Is Mediated by Small Molecules

To affirm the metabolic nature of the yeast-LAB interaction, we first tested whether yeast-conditioned medium (cell-free filtrate of yeast culture) was sufficient to support bacterial growth. Indeed, both *L. lactis* and *L. plantarum* grew well in the conditioned medium, recapitulating the co-culture effect (Figure 1E). This result shows that: (1) yeast produce diffusible factor(s) that sustain the LAB growth, (2) direct physical contact between yeast and LAB is not required for the interaction, and (3) release of growth-promoting factor(s) is an inherent property of yeast monoculture and does not need to be induced by bacteria. The effect of yeast on LAB was not due to change in pH. Furthermore, the factor(s) in the yeast-conditioned medium that enable growth of LAB were found to be resistant to protease treatment, remained in the flow-through fraction in anion exchange or reverse-phase chromatography, passed through a 3 kDa filter, could be precipitated with acetone, and partially extracted with acetonitrile but not with low polarity organic solvents. These characteristics hint at small, hydrophilic metabolites as mediators of the yeast-LAB interaction.

### A Genome-Scale Community Metabolic Model Predicts Amino Acid Cross-Feeding

To further investigate the nature of possible exchanged metabolites, we simulated metabolite exchange in our three-species community using a genome-scale metabolic modeling approach (Zelezniak et al., 2015) (STAR Methods). In brief, we combined the three-species-level metabolic models into a community model wherein the availability of the nutrients from the environment was restricted according to the CDM35 composition. The community model was then used to enumerate, using mixed-integer linear programming, all possible metabolite exchanges that could sustain the growth of all three species. Confirming experimental observations, two LAB models were unable to grow (i.e., to produce the building blocks and the co-factors necessary for cell growth) without metabolic support from the yeast. In particular, the community model predicted a flow of amino acids (glutamine, glutamate, proline, and phenylalanine) from yeast to bacteria (Figure S2). In addition, 2-oxoglutarate, a co-substrate of various transamination reactions in amino acid biosynthesis, and the polyamine spermidine, were predicted in the case of *L. plantarum*. These results suggest that nitrogenous compounds, in particular amino acids, as a candidate class of exchanged metabolites.

### Exo-Metabolome Dynamics Reveals Multi-Component Cross-Feeding

**Figure 2:**
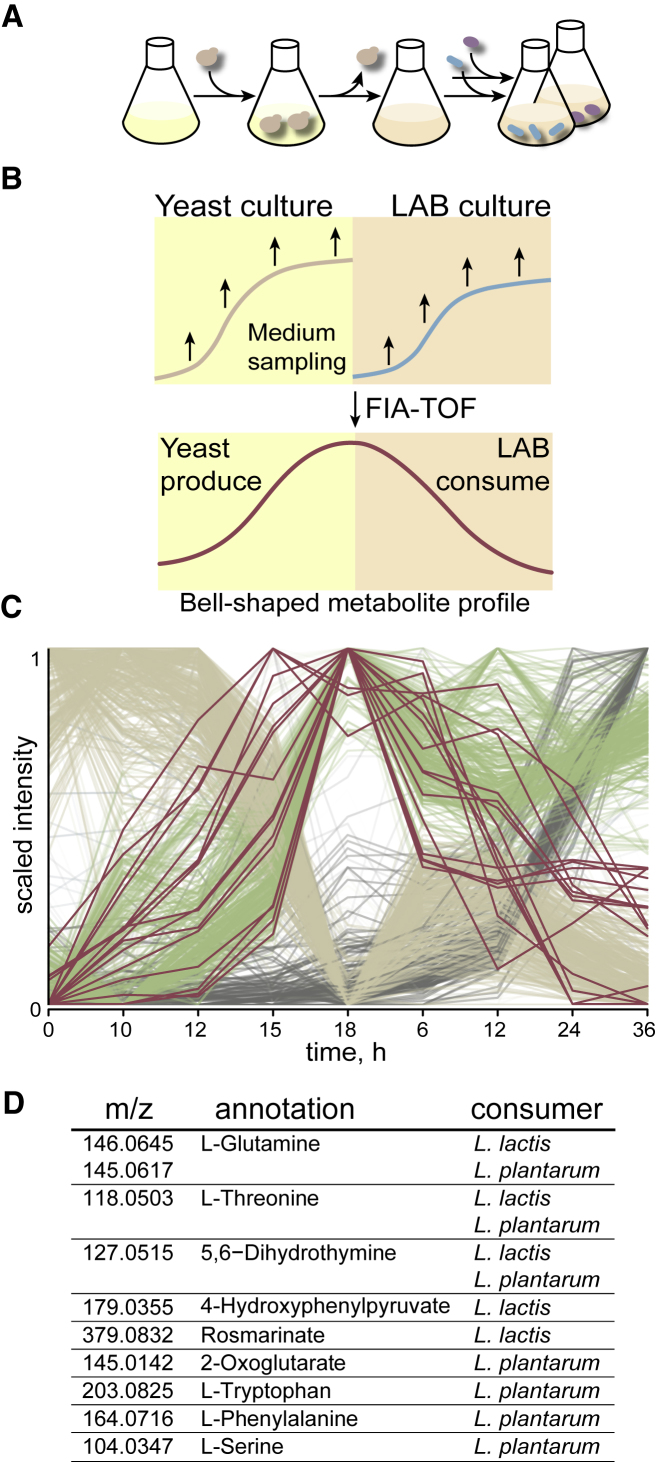
Identification of Yeast Secretome Components that Enable Growth of LAB (A) Conditioned medium assay design. (B) Untargeted metabolomics workflow (flow injection analysis time of flight [FIA-TOF] mass spectrometry) as applied to the conditioned medium assay. (C) Exo-metabolome dynamics of *S. cerevisiae* and *L. plantarum* revealed by untargeted metabolomics. Shown are the groups of ions with distinct profile shape. Cluster of metabolites potentially cross-fed from yeast to bacteria (bell-shaped curves) is highlighted in red. Data for one sample are shown, see also Figure S3 for summary statistics. (D) Annotated metabolites produced by *S. cerevisiae* and consumed by LAB (with at least 2-fold change in both accumulation and decrease). See also Figure S3. Note that ion annotation based on accurate mass may be ambiguous; see Table S2 for complete annotation.

To experimentally pinpoint exchanged metabolites, we combined the above-described conditioned medium setup with an untargeted mass spectrometry approach (Fuhrer et al., 2011). Use of conditioned medium allowed for the accumulation of cross-fed molecules before uptake by LAB, circumventing difficulties in detecting low-concentration transiently exchanged metabolites. In conjunction, the mass spectrometry method allowed us to profile the dynamics of a large number of secreted/uptaken compounds. Samples of supernatant were taken at multiple time points during the conditioning of the medium with yeast and subsequently during the growth of LAB in the conditioned medium (Figures 2A and 2B). Mass spectrometry analysis revealed diverse, dynamic profiles for hundreds of ions, characterizing the complex yeast-LAB exo-metabolome (Figure 2C). In total, 10,620 metabolite ions were detected that could be putatively annotated to 2,225 metabolites based on exact mass (STAR Methods, Table S2). Metabolites that accumulate with the growth of *S. cerevisiae* and become depleted during bacterial growth, thus exhibiting characteristic bell-shaped profiles (red cluster in Figure 2C), were selected as candidates mediating yeast-LAB interaction. This cluster shows many candidate exchanged compounds, among which 9 ions for *L. plantarum* and 11 for *L. lactis* showed over 2-fold change during both accumulation and depletion (Figures 2D and S3). Many of these were annotated as amino acids, suggesting cross-feeding of glutamine and threonine to *L. lactis*, and glutamine, threonine, phenylalanine, tryptophan, and serine to *L. plantarum*. Cross-feeding of 2-oxoglutarate was also observed, as predicted by metabolic modeling. Overall, untargeted exo-metabolomics suggests that LAB dependency on yeast is mediated by cross-feeding of multiple components. It also marks the candidate metabolites for further analysis for confirming their identity, quantification, and ability to support LAB growth.

### Amino Acids Are Major Agents of Yeast-LAB Interaction

**Figure 3:**
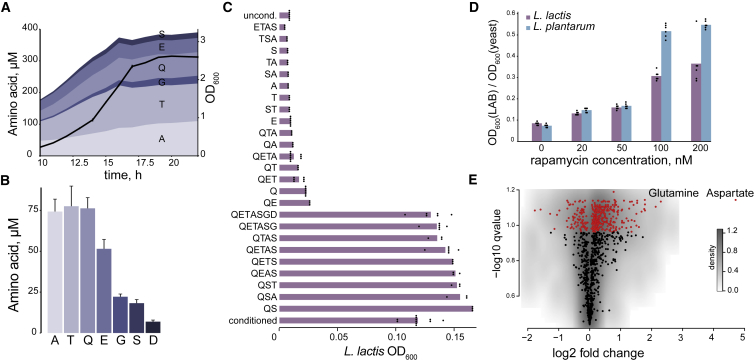
Amino Acids Secreted by *S. cerevisiae* and Rapamycin Effect (A) Dynamics of secreted amino acids in *S. cerevisiae* exo-metabolome. Black line shows yeast cell density with error bars representing means ± SD (n = 4 biological replicates). See also Figure S4. (B) Concentration of secreted amino acids in yeast-conditioned medium (at optical density at 600 nm [OD_600_] ∼ 1). Data shown as mean ± SD (n = 4 biological replicates). (C) Effect of supplementing identified amino acids (in respective concentrations) to naive medium on the growth of *L. lactis*. Data shown as mean and pooled technical replicates of three biological replicates. See Figure S5 for *L. plantarum* data. (D) Effect of culturing yeast in presence of rapamycin on LAB growth in respective conditioned media. Data shown as mean and pooled technical replicates of three biological replicates. (E) Changes in exo-metabolome of yeast-conditioned medium when cultured in the presence of rapamycin, estimated by untargeted metabolomics. Data normalized by the areas under the curve of corresponding yeast cultures. Red color indicates q values (false discovery rate-corrected t test-derived p values) < 0.1 (n = 3 biological replicates). See Figure S6 for metabolites cross-fed from yeast to bacteria (bell-shaped profiles) in the presence of rapamycin.

Following the indications of candidate exchanged metabolites by exo-metabolome dynamics, we quantified amino acids secreted by yeast using a targeted liquid chromatography-tandem mass spectrometry method (Mülleder et al., 2016a). Concentrations of extracellular amino acids in the yeast culture increased proportionally to the cell density and stabilized in stationary phase (Figure 3A). In accord, the conditioned medium prepared at different growth stages supported bacterial growth, even at the early growth stage (Figure S4). These observations are consistent with the growth-promoting components being produced by metabolically active cells rather than released due to cell lysis. In addition, the rather low fraction of dead/damaged yeast cells (around 0.04%, STAR Methods) cannot explain the observed concentration of amino acids in the conditioned medium (STAR Methods), consistent with previous studies showing that yeast exo-metabolome is not a result of the cell lysis (Campbell et al., 2015, Paczia et al., 2012). The most abundant amino acids accumulating in the yeast-conditioned medium were threonine, glutamine, alanine, glutamate, serine, and glycine (Figure 3B). Targeted metabolomics results thus agree well with the untargeted exo-metabolomics, confirming amino acid exchange and in particular cross-feeding of glutamine and glutamate.

Further, we asked if supplementing un-conditioned medium with these amino acids, supplied in the observed concentrations, could reproduce the growth-enabling effect of the conditioned medium. Indeed, the identified amino acids fully restored growth of *L. lactis* to the levels observed in the conditioned medium (Figure 3C). In particular, glutamine and serine were both required and sufficient for *L. lactis* growth, demonstrating synergy. The slightly higher growth of *L. lactis* in the supplemented media, in comparison with the conditioned medium (Figure 3C), is likely due to depletion of the medium by yeast, and/or inhibiting effects of other secreted compounds like ethanol. Thus additional metabolites in the yeast-conditioned medium may also contribute to *L. lactis* growth as indicated by exo-metabolome dynamics.

In the case of *L. plantarum*, supplementation of quantified amino acids to the CDM35 medium had only marginal effect on its growth (Figure S5). However, many other metabolites besides identified amino acids might be supporting *L. plantarum* growth. The synergistic nature of such a multi-component cross-feeding can make it challenging to characterize, since removal of even one metabolite will abolish all the effect. To explore which yeast exo-metabolites, besides amino acids, could be involved in sustaining growth of *L. plantarum*, we tested a number of compounds detected in the conditioned medium (2-oxoglutarate and putrescine) (Table S3), identified by untargeted metabolomics (tryptophan, phenylalanine, and 2-oxoglutarate), predicted by modeling (phenylalanine and 2-oxoglutarate), or hypothesized based on known LAB physiology (nucleobases and ascorbate) (Figure S5). Indeed, several combinations of the predicted/hypothesized compounds were found to be effective in synergy with amino acids identified in the conditioned medium. Although the specific tested metabolites were not identified in the conditioned medium, the unknown cross-fed compounds may be nutritional analogs of the identified effectors, e.g., short peptides substituting for phenylalanine and tryptophan.

### TORC1 Pathway and NCR Genes Impact Yeast-LAB Interaction

Next we sought to explore the regulatory processes that prompt yeast to secrete nutrients enabling growth of LAB. Since cellular metabolism is often regulated in response to nutritional cues, we hypothesized involvement of the major nutrient, sensing controller of eukaryotic cells, TORC1 (target of rapamycin complex 1). Indeed, in the presence of rapamycin, a specific inhibitor of TORC1, the positive effect of yeast-conditioned medium on both *L. lactis* and *L. plantarum* was boosted up to 5-fold (Figure 3D). Consistently, the untargeted exo-metabolome analysis showed increased secretion of multiple metabolites in the presence of rapamycin, especially of the amino acids glutamine and aspartate (Figure 3E). The exchanged metabolites, detected as bell-shaped profiles in the untargeted analysis, were also mostly the same (Figure S6).

**Figure 4:**
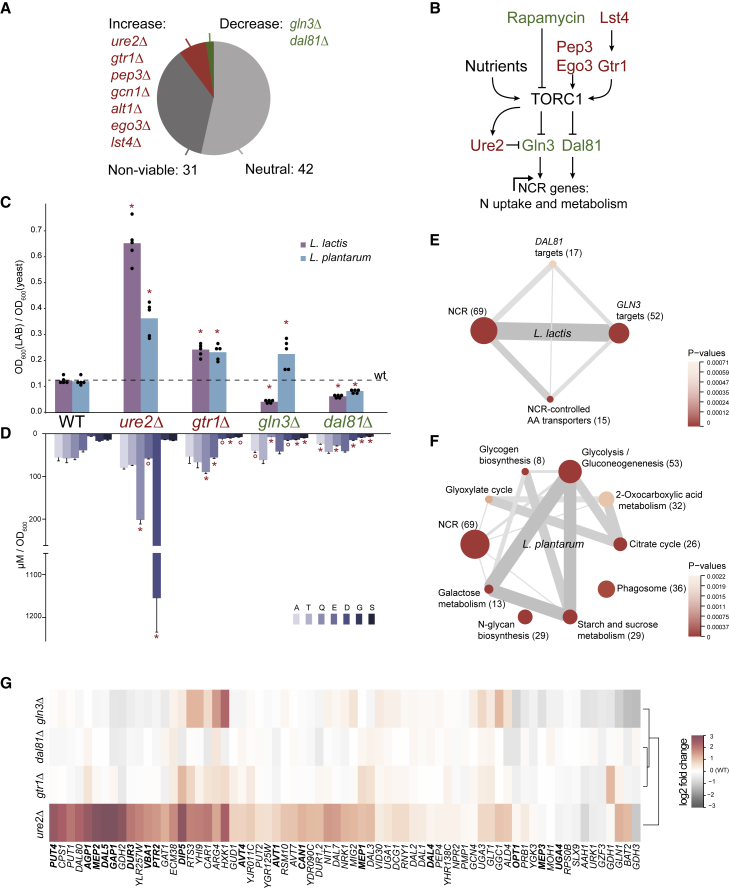
Yeast Knockout Strains Indicate NCR-Sensitive Genes as Regulators of Interaction with LAB (A) Effect of TORC1 pathway-related single-gene knockout strains on LAB growth (relative to the wild-type). See also Table S5. (B) Interactions of NCR/TORC1 regulators that affect yeast-LAB interactions, as per literature review. Green color highlights proteins, of which the corresponding gene knockout downregulates the effect of yeast on LAB, and the red color shows effectors whose absence has a positive effect. (C) Selected *S. cerevisiae* knockout mutants with altered effect on LAB growth (compared with the wild-type). ^∗^p < 0.01. n = 5 biological replicates. (D) Concentrations of amino acids in exo-metabolome of knockout yeast strains. ^∗^p < 0.01, °p < 0.05. Data shown as mean ± SD (n = 4 biological replicates). See also Figure S7. (E) Gene sets enriched for genes correlating with *L. lactis* growth (across the four selected knockout strains and the wild-type). Shown are groups with enrichment p < 0.01 (see the STAR Methods). Number of genes in each set is given in parentheses. (F) Same as in (E) for *L. plantarum*. Shown are top ten non-redundant groups with enrichment p < 0.01. (G) Expression of NCR genes (Ljungdahl and Daignan-Fornier, 2012) in selected knockout strains (log2 fold change relative to the wild-type, n=4 biological replicates). Amino acid and peptide transporters are shown in bold.

In response to nutritional cues, TORC1 regulates a broad range of cellular processes, including growth, autophagy, protein synthesis, and nitrogen metabolism (Broach, 2012, Conrad et al., 2014, Loewith and Hall, 2011, Zaman et al., 2008). To clarify which TORC1-related processes are involved in the yeast-LAB interaction, we tested 82 single gene knockout strains of *S. cerevisiae* for their ability to support LAB. These genes were selected to cover the main effectors of TORC1 signaling, both upstream and downstream of TORC1 complex (Conrad et al., 2014, De Virgilio and Loewith, 2006, Fayyadkazan et al., 2014, Loewith and Hall, 2011) (Table S5). These included transcription factors and other regulatory proteins, as well as enzymes and transporters involved in amino acid metabolism. To be able to cultivate yeast without any artificial/synthetic dependency on amino acid supplementation, and to avoid the physiological impact of auxotrophic markers (Alam et al., 2016), all strains were obtained from a prototrophic deletion library (Mülleder et al., 2012). Among the 51 knockout mutants that grew in CDM35, seven considerably increased and two reduced the LAB growth (Figure 4A, Table S5). These nine effectors are transcription factors or signaling proteins, suggesting that yeast-LAB interaction is a complex phenotype, perturbation of which requires a pleiotropic effect. Knockout strains increasing the LAB growth (*ure2Δ*, *gtr1Δ*, *pep3Δ*, *gcn1Δ*, *alt1Δ*, *lst4Δ*, and *ego3Δ*) affected both *L. lactis* and *L. plantarum* similarly. Deletion of either of the two transcription factor encoding genes (*GLN3* or *DAL81*) significantly reduced the growth of *L. lactis* (p < 0.001), but only deletion of *DAL81* reduced *L. plantarum* growth (p < 0.001). This difference, again, suggests that the two LAB species depend on yeast through different sets of metabolites.

Notably, most of the genes whose deletions increase LAB growth encode direct or indirect repressors of Gln3p and Dal81p (Conrad et al., 2014, Loewith and Hall, 2011, Smets et al., 2010) (Figure 4B). Both Gln3p and Dal81p are among the key regulators of nitrogen catabolite repression (NCR), a process responsible for selective utilization of nitrogen sources (Cooper et al., 1990, Cooper, 2002, Hofman-Bang, 1999, Rai et al., 2013). Whenever a preferred source of nitrogen is present (such as glutamine), yeast prioritizes its consumption and shuts down the uptake and catabolism of the other nitrogen sources. In this state, NCR-sensitive genes are repressed. Conversely, when yeast is grown on unfavorable nitrogen source (such as proline), cells activate NCR genes to assimilate the available nitrogen compounds through alternative metabolic routes. Gln3p and Dal81p positively regulate this process through induction of permeases, catabolic enzymes, and other transcription factors (Abdel-Sater et al., 2004, André et al., 1995, Bricmont et al., 1991, Cardillo et al., 2010). Yeast mutant phenotypes indicate that the NCR pathway also mediates metabolite secretion that creates a niche for LAB.

### Yeast Strains with Differential Support to LAB Differ in Amino Acid Secretion

To better understand the regulation of yeast metabolite secretion, we compared amino acid secretion profiles of four yeast knockout strains: two reducing (*gln3Δ* and *dal81Δ*), and two increasing (*ure2Δ* and *gtr1Δ*) LAB growth. First, to ensure that the results obtained with the systematically created library strains are not artificial, we constructed these four strains in a more stable, prototrophic S90 background (STAR Methods). These newly created deletion mutants confirmed the impact of the corresponding genes on LAB growth (Figure 4C). We also checked that the mutant effects on bacterial growth are not confounded by differences in their growth rates (Figure S7A) and the degree of cell damage/lysis remains low (maximum of 0.22%; Figures S7B and S7C).

The effect of the yeast knockout strains on *L. lactis* growth correlates well with the total amino acid concentration in the corresponding conditioned media (rho = 0.9, p = 0.0416), with a prominent role of glutamine (rho = 1, p = 0.0083) (Figure 4D). In the case of *L. plantarum*, reduced amino acid concentrations for *gln3Δ* is at odds with an increase in its growth. This further points to the involvement of exchanged metabolites other than amino acids. The *ure2Δ* and *gtr1Δ* mutants excrete more amino acids than the wild-type strain, with an especially dramatic increase in aspartate and glutamine secretion by the *ure2Δ* strain. Consistent with the regulatory relation between Ure2p and TORC1, the same metabolites were prominently increased in the yeast exo-metabolome upon rapamycin treatment (Figure 3E). Furthermore, *ure2Δ* mutant was found, in a recent genome-wide screening of intracellular amino acid levels (Mülleder et al., 2016b), to be the top glutamine accumulator (3.4-fold increase over the median, p < 1.6 × 10^−69^).

Overall changes in the secretome of knockout strains show that action of positive regulators of NCR-sensitive genes (*GLN3* and *DAL81*) is associated with increased amino acids secretion, and, accordingly, inhibitors of NCR (*URE2* and *GTR1*) appear to downregulate amino acids secretion. In addition, we identified gene sets with expression positively associated with growth-enabling effect of yeast on LAB. To do so, we performed pathway enrichment analysis for genes expressed in yeast deletion strains that highly correlate with bacterial final optical density at 600 nm in the corresponding conditioned media. Indeed, the expression levels of the NCR-sensitive genes were among the top scoring for positive correlation with the growth of *L. lactis* as well as *L. plantarum* (Figures 4E and 4F). These genes showed highest expression levels in the *ure2Δ* strain, which secretes the largest amounts of amino acids and sustains the highest counts of symbiotic LAB (Figure 4G). Interestingly, expression of a subset of yeast amino acid transporters controlled by NCR (according to Ljungdahl and Daignan-Fornier, 2012) was also found to be correlated with *L. lactis* growth. Secretion of amino acids that enables *L. lactis* growth thus appears to be regulated via NCR pathways.

### Plentiful Nitrogen Sources Are Needed for Amino Acid Secretion by Yeast

**Figure 5:**
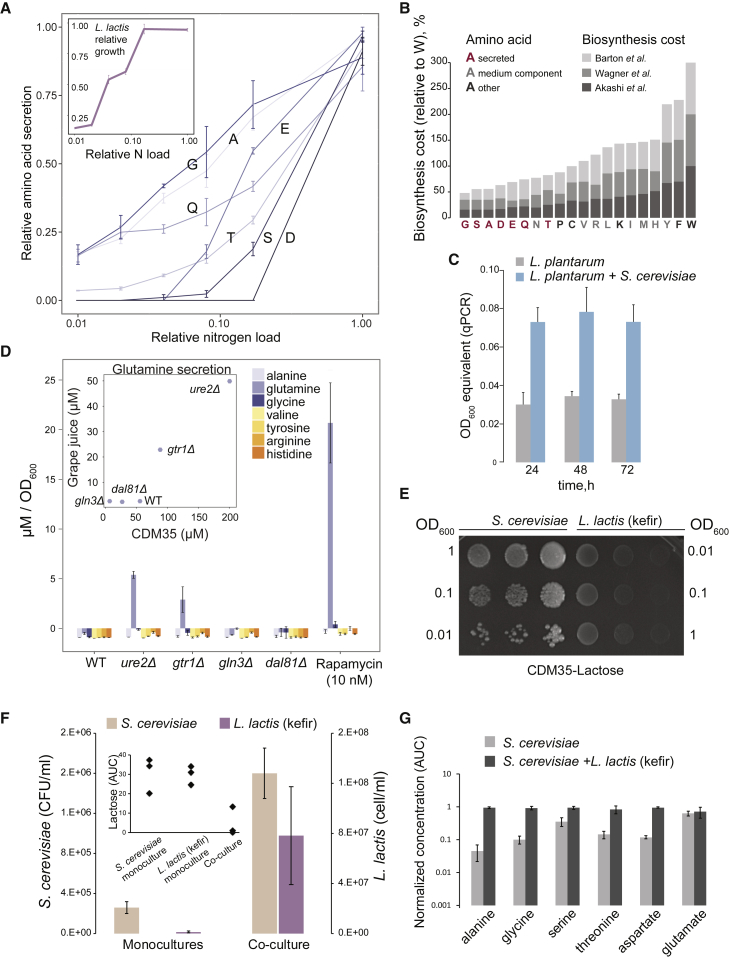
Niche Creation through Nitrogen Overflow and Emergence of Mutualism (A) Amino acid secretion by yeast is proportional to total nitrogen load. Shown are relative secretion levels at different degrees of total nitrogen content. See Table S7 for amino acid composition. (B) Secreted amino acids have the lowest cost of biosynthesis. Stacked bars represent different cost metrics: unitless costs based on flux balanced changes in uptakes (Barton et al., 2010, top), respiratory energetic cost (Wagner, 2005, middle), and energetic cost of biosynthesis, including biosynthesis of precursors (Akashi and Gojobori, 2002, bottom). (C) *S. cerevisiae* S90 shows positive effect on *L. plantarum* growth when co-cultured in grape juice. Note: grape juice pH did not affect yeast-LAB interaction (Figure S9). (D) Amino acid uptake/secretion by yeast in grape juice in response to rapamycin and NCR/TORC1 pathway mutations. Inset: glutamine secretion by different yeast strains in CDM35 versus grape juice. See also Figures S8 and S10. (E) Mutualistic growth of yeast and *L. lactis* kefir isolate in CDM35-Lactose. OD_600_ values refer to the seed cultures. (F) Mutualistic relation between yeast and *L. lactis* (kefir) is also evident in liquid cultures. The inset shows residual lactose in mono and co-cultures. *L. lactis* (kefir) cell counts are based on flow cytometry (STAR Methods). (G) Amino acid concentration in yeast monocultures and co-cultures with *L. lactis* (kefir) in CDM35-lactose. Data shown as mean ± SD of three independent replicates in (A, C, D, F, and G).

We hypothesized that the secretion of amino acids by yeast would depend on total nitrogen concentration in the medium. To check this, we proportionally reduced the concentration of nitrogen compounds in CDM35 and measured amino acids in these conditioned media (see Table S7). Indeed, we found that the total amino acid secretion levels decreased proportionally to the reduction in the available nitrogen (Figure 5A). Yeast growth was not affected by the change in nitrogen availability in this range (see Table S7). We also observed that the secreted amino acids are the ones with the lowest biosynthetic costs (Figure 5B). All these observations indicate overflow metabolism, possibly employed as a means to dispose of excess nitrogen in the form of “cheap” amino acids.

### Yeast-LAB Symbiosis in Ecological Context

We next investigated the relevance of described metabolic dependency between yeast and LAB in their natural habitat. We tested interaction between *S. cerevisiae* and *L. plantarum* in a grape juice, a natural medium wherein these two species co-occur, e.g., in certain wine fermentations (Jouhten et al., 2016). Indeed, the yeast-LAB interaction observed in CDM35 was evident also in grape juice (Figure 5C). Consistent with the criteria of high nitrogen load necessary for overflow metabolism, many amino acids were detected in natural grape juice in concentrations comparable with those in CDM35 (Table S8; Figure S8). Furthermore, rapamycin and yeast NCR mutants affect *L. plantarum* growth in grape juice similarly to CDM35 conditions (Figure S10). The highest growth boost for *L. plantarum* is provided by rapamycin addition and *ure2Δ*, followed by *gtr1Δ* and *gln3Δ* mutants. In the case of *dal81Δ* mutant, the observed growth reduction of *L. plantarum* in CDM35 is likely masked in grape juice because *L. plantarum* can grow in grape juice, but not in CDM35, to some extent on its own. Glutamine secretion by *gln3Δ*, *dal81Δ*, *ure2Δ*, and *gtr1Δ* mutants and in response to the rapamycin treatment indicates that TORC1/NCR-associated mechanisms described for CDM35 also act in the grape juice environment (Figure 5D). Moreover, we tested yeast isolates from wineries and kefir, all of which enabled growth of *L. lactis* and *L. plantarum*, showing that secretion of LAB-beneficial metabolites is widely conserved among yeasts (Figure S11).

Metabolic dependency between yeast and LAB appears to be unidirectional in both CDM35 and grape juice; the benefit for yeast being balancing its own metabolism independent of LAB. We wondered whether amino acid secretion by yeast could also form a basis for establishment of a bidirectional nutrient dependency. Given the co-existence of yeast and LAB in several fermented milk drinks (Prado et al., 2015, Tamang et al., 2016), we addressed this question by using an *L. lactis* strain isolated from kefir and replacing glucose in CDM35 with lactose, primary milk sugar. We hypothesized that, while *L. lactis (kefir)* would depend on yeast for nitrogen as before, yeast would depend on *L. lactis (kefir)* for carbon due to its inability to metabolize lactose. Confirming this, while the dependency in CDM35-glucose medium was unidirectional as expected (Figure S12), mutual dependency between yeast and *L. lactis* (*kefir*) was evident in both solid and liquid CDM35-lactose medium (Figures 5E and 5F). Growth of either species, as well as lactose utilization, was observed only in co-cultures (Figure 5F), and the co-culture supernatant showed, compared with the yeast monocultures, severalfold increase in amino acids that were also secreted in CDM35-glucose (Figure 5G). These results demonstrate that unidirectional metabolic dependency can develop into mutualistic cross-feeding with a single nutrient change.

## Discussion

Challenges of measuring metabolite exchange in microbial communities limit the number of interactions documented to date; even in synthetic communities known interactions typically constitute one or two exchanged metabolites, often inferred without direct metabolite measurements (Ponomarova and Patil, 2015). Our results bring insights into the complex multi-metabolite cross-feeding in rich environments. By combining metabolomics and genetics, we could discover the details and an unexpected complexity of a naturally established multi-metabolite cross-feeding between yeast and LAB.

The identified cross-feeding interactions show that community metabolic modeling can provide a valuable supplement to the experimental data. Although the overall simulation result was incomplete (serine exchange was missed), and glutamine and glutamate were incorrectly predicted to be of equal value, prediction of the exchange of nitrogenous metabolites as a class was correct and allowed to prioritize corresponding hits from the untargeted metabolomics in a targeted follow-up analysis. The metabolomics data from our study could be useful to improve the accuracy of the individual LAB models as well as the yeast-LAB community model.

Beyond discovering metabolic interactions, this study provides insights into regulatory processes prompting yeast to secrete metabolites. Our results suggest a model where diverse and abundant nitrogen sources in the environment cause a potentially damaging imbalanced nutrient uptake, which prompts yeast, via NCR-sensitive pathways, to secrete excess nitrogen as amino acids that coincidentally benefit the LAB. Amino acid secretion, or overflow, in yeast indeed can be caused by the excess uptake of certain amino acids (Velasco et al., 2004) or peptides (Melnykov, 2016). Furthermore, yeast can also secrete amino acids in media without supplemented amino acids, albeit in relatively small quantities (Paczia et al., 2012). Nitrogen excess, especially under other nutrient limitation, has been shown to elicit negative effects on yeast growth and cell viability (Chen and Kaiser, 2002, Hess et al., 2006, Risinger et al., 2006, Tesniere et al., 2013). Consequently, nitrogenous compounds such as amino acids are secreted as a potential mechanism to dispose of excess and/or potentially toxic levels of intracellular nitrogen (Hess et al., 2006, Tesniere et al., 2013). Since yeasts prefer to uptake amino acids from the medium instead of synthesizing them (Campbell et al., 2015), a variety of amino acids in the medium can compete for the transport by general permeases, leading to imbalanced uptake. In agreement with our data, general amino acid permease Gap1p and TORC1 were previously shown to contribute to nitrogen toxicity (Chen and Kaiser, 2002, Risinger et al., 2006, Santos et al., 2012). Indeed, we find that, among all the transporters, the expression of GAP1p is the best correlating with *L. lactis* growth across different mutants (rho = 1, p = 0.017). This also supports that the amino acid secretion observed in our study is a regulated phenomenon. Supporting the detoxification theory from a cellular economy point of view, we observe that biosynthetic costs (Akashi and Gojobori, 2002, Barton et al., 2010) are among the lowest for the observed overflow amino acids.

**Figure 6:**
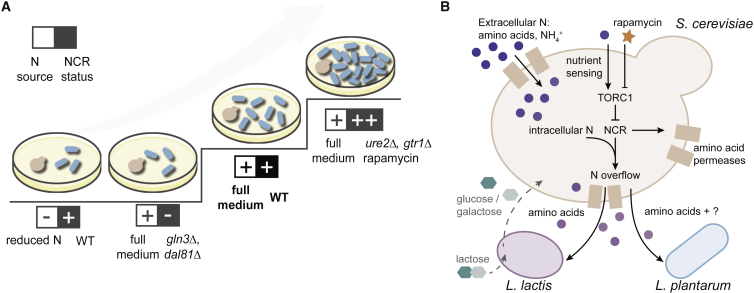
Metabolic Environment and Cellular Regulation Jointly Determine Niche Creation by Yeast (A) Factors that are jointly required for yeast-LAB interaction: diverse and plentiful nitrogen sources in the medium and activity of NCR-sensitive genes, the latter being higher in *ure2Δ*, *gtr1Δ*, and rapamycin-treated cells and lower in *gln3Δ* and *dal81Δ* strains. (B) Metabolite overflow as a result of diverse nitrogen sources processed through NCR-regulated metabolic processes. The overflow amino acids enable survival of LAB. *L. lactis* in turn reciprocates when glucose is substituted for lactose.

Two components emerge as being essential for amino acid overflow: presence of abundant nitrogen sources and expression of NCR-regulated genes. The former provides the “raw material,” and the latter incorporates it into central metabolism. Joint action of these two factors, in varying strength, creates a gradient of interaction phenotypes (Figure 6A). Both factors are present when wild-type yeast is grown in CDM35: multiple amino acids and basal expression of NCR genes. If any of the two driving forces gets diminished, either richness of the medium (by reducing nitrogen sources) or NCR (through knockout of *DAL81* or *GLN3*), fewer nutrients are being released. In contrast, upregulating NCR genes with rapamycin, or by deleting genes coding for transcription factors Ure2p or Gtr1p, while keeping the plentiful nitrogen sources, allows increasing the metabolite excretion. Abundant nitrogen sources are indeed also present in ecological niches of yeast such as grape juice; wherein we show that mechanism of NCR-associated nitrogen overflow is also at play and benefits LAB. Furthermore, processes such as nitrogen overflow, where waste products of one organism become essential growth-limiting nutrients for the other, can be viewed as first steps in evolution of mutualism. We demonstrate this by showing how nitrogen overflow by yeast contributes to ready emergence of mutualism with *L. lactis* with a single nutrient change in the environment.

The mechanistic picture of nitrogen overflow provided by our study (Figure 6B) suggests that nutrient balancing and NCR pathways could be exploited to regulate species dynamics in numerous yeast-LAB communities. LAB are fastidious organisms (Hayek and Ibrahim, 2013, Van Niel and Hahn-Hägerdal, 1999, Wegkamp et al., 2010, Zhang et al., 2009). Even habitats that are overwhelmingly nutritious for yeasts can be too poor to support LAB growth. Yet, as we show here, by-products of yeast metabolism can open a metabolic niche creating a lasting ecological effect on LAB. While yeast-LAB symbiosis is welcome in many fermented products, such as kefir and kimchi, LAB are notorious for contaminating wine, beer and bio-ethanol fermentations (Carvalho-Netto et al., 2015, Jespersen and Jakobsen, 1996, Vaughan et al., 2005). Demonstrating the relevance of yeast-LAB interaction for wild isolates and grape juice environment brings us one step closer to controlling interspecies interactions *in situ*. In particular, the nitrogen overflow metabolism appears to be one of the forces that, following fluctuations of nutrient availability in dynamic natural systems, can make or break symbiotic interspecies relationship.

Our yeast-LAB community showcases a complex eukaryote-prokaryote cross-feeding in an instantaneously established and stable symbiosis. While this community makes a genetically and environmentally tunable model system for studying emergence and dynamics of interspecies interactions, the experimental approach bears a high potential for discovering metabolic ties in other microbial consortia. In summary, our findings uncover the primary process of metabolic niche creation in molecular detail and show how survival of one species can be driven by metabolic idiosyncrasies of the other.

## STAR★Methods

### Key Resources Table

**Table:** 

REAGENT or RESOURCE	SOURCE	IDENTIFIER
**Bacterial and Virus Strains**		

WCFS1, *Lactobacillus plantarum*	BCCM/LMG (http://bccm.belspo.be/)	LMG 9211
IL1403, *Lactococcus lactis* ssp. *lactis*	INRA	LMG 6890
*Lactococcus lactis* 17, isolated from kefir grains.	K. R. Patil Laboratory -Blasche S.	SB17

**Chemicals, Peptides, and Recombinant Proteins**		

Rapamycin	Sigma Aldrich	Cat#R8781
GM-17 medium (M-17 broth supplemented with 5% glucose)	Sigma Aldrich	Cat#56156
MRS broth	Sigma Aldrich	Cat#69966
Chemically defined medium 35 (CDM35) described in Table S1	This paper	N/A
PEG 200	Sigma Aldrich	Cat#88440
Amino acids (analytical standard)	Sigma Aldrich	Cat# LAA21
Cycloheximide solution	Sigma Aldrich	Cat#18079
Propidium Iodide, ≥94.0% (HPLC)	Sigma Aldrich	Cat#P4170
G-418 Disulphate	Formedium Ltd.	Cat#G4185
Ribitol (Adonitol), 99%	Alfa Aesar, UK	Cat#L03253
Pyridine, HPLC Grade, 99.5+%	Alfa Aesar, UK	Cat#22905
N-Methyl-N-(trimethylsilyl)trifluoroacetamide, 97% (MSTFA)	Alfa Aesar, UK	Cat#A13141
Methoxyamine hydrochloride, 98+% (MeOx)	Alfa Aesar, UK	Cat#A19188

**Critical Commercial Assays**		

LIVE/DEAD FungaLight Yeast Viability Kit	Thermo Fisher Scientific-Invitrogen™	Cat#L34952
Amicon Ultra-0.5 Centrifugal Filter Unit with Ultracel-3 membrane	EMD Millipore	Cat#UFC500396
RNeasy Mini Kit	QIAgen	Cat# 74104
CountBright Absolute Counting Beads	Thermo Fisher Scientific	Cat#C36950
SYBR Green PCR Master Mix	Thermo Fisher Scientific- Applied Biosystems	Cat#4309155
AccQ-Tag Ultra Chemistry Kit	Waters	Cat#176001235

**Deposited Data**		

Transcriptome dataset (*ure2Δ*, *gtr1Δ*, *dal81Δ*, *gln3Δ* and the wild type S. cerevisiae of S90 genetic background in CDM35)	This paper	http://www.ebi.ac.uk/arrayexpress/ Accession number: E-MTAB-4651
Updated *L. lactis* genome-scale metabolic model iAOP358	This paper	https://www.patil.embl.de/models/
Raw untargeted metabolomics dataset (metabolite dynamics in yeast and LAB conditioned CDM35)	This paper; Mendeley Data	http://dx.doi.org/10.17632/r5bx5cg9y6.1
Targeted metabolomics dataset (amino acids in yeast conditioned CDM35)	This paper; Mendeley Data	http://dx.doi.org/10.17632/h2fvn2mpp3.1

**Experimental Models: Organisms/Strains**		

S90, *Saccharomyces cerevisiae* (*MATα GAL2*)	(Steinmetz et al., 2002)	S90
Prototrophic *Saccharomyces cerevisiae* gene deletion collection.	(Mülleder et al., 2012)	http://www.euroscarf.de
PRICVV29, *Torulaspora delbrueckii (Lindner) Lindner 1904*.	Ramón González Laboratory (Logrono, Spain)	CECT 1880
PRICVV678, *Torulaspora delbrueckii (Lindner) Lindner*.	Ramón González Laboratory (Logrono, Spain)	ATCC 10662/ CBS 1146
*Candida californica 48,* isolated from kefir grains.	K. R. Patil Laboratory -Blasche S.	SB48
*Kluyveromyces marxianus 72,* isolated from kefir grains.	K. R. Patil Laboratory -Blasche S.	SB72
*Kazachstania exigua 178,* isolated from kefir grains.	K. R. Patil Laboratory -Blasche S.	SB178
*Rhodotorula mucilaginosa 353*, isolated from kefir grains.	K. R. Patil Laboratory -Blasche S.	SB353
*Saccharomyces unispora 162*, isolated from kefir grains.	K. R. Patil Laboratory -Blasche S.	SB162
PRICVV50, *Saccharomyces cerevisiae bayanus*, wine yeast.	Ramón González Laboratory (Logrono, Spain)	Lalvin EC-1118
PRICVV55, *Saccharomyces cerevisiae bayanus*, wine yeast.	Ramón González Laboratory (Logrono, Spain)	Lalvin T73
*dal81Δ* (S90, *dal81::kanMX4*).	This paper	NG15
*gln3Δ* (S90 *gln3::kanMX4*).	This paper	NG16
*gtr1Δ* (S90 *gtr1::kanMX4*).	This paper	NG17
*ure2Δ* (S90 *ure2::kanMX4*).	This paper	NG18

**Oligonucleotides**		

Primers used in this study are listed in Table S6.	This paper	N/A

**Software and Algorithms**		

R: A Language for Data Analysis and Graphics, version 3.2	http://www.r-project.org	N/A
piano	(Varemo et al., 2013)	N/A
grofit	(Kahm et al., 2010)	N/A
FastQC	https://www.bioinformatics.babraham.ac.uk/projects/fastqc/	N/A
FaQCs	(Lo and Chain, 2014)	N/A
TopHat	(Trapnell et al., 2009)	N/A
HTSeq-count	(Anders et al., 2015)	N/A
DESeq2	(Anders and Huber, 2010)	N/A
MassHunter software suite	Agilent Technologies	N/A
SMETANA framework	(Zelezniak et al., 2015)	N/A

**Other**		

*Saccharomyces cerevisiae* genome-scale metabolic model iAZ900	(Zomorrodi and Maranas, 2010)	N/A
*Lactococcus lactis* genome-scale metabolic model iAO358	(Oliveira et al., 2005)	N/A
Modified iAOP358 model	This paper. https://www.patil.embl.de/media/models/ponomarova/iAOP358.bioopt	N/A
*Lactobacillus plantarum* genome-scale metabolic model	(Teusink et al., 2006)	N/A
*Lactococcus lactis* genome-scale metabolic model (automated reconstruction)	(Henry et al., 2010)	N/A
*Lactobacillus plantarum* genome-scale metabolic model (automated reconstruction)	(Henry et al., 2010)	N/A

### Contact for Reagent and Resource Sharing

Further information and requests for reagents may be directed to, and will be fulfilled by the corresponding author, Dr. K. R. Patil (patil@embl.de).

### Experimental Model and Subject Details

#### Strains, Media and Growth Conditions

Prototrophic strains of *Saccharomyces cerevisiae* S90, *Lactobacillus plantarum* WCFS1, and *Lactococcus lactis* subsp. *lactis* IL1403 were pre-cultured using YPAD, MRS, and GM-17 medium (M-17 supplemented with 5% glucose) respectively. Kefir isolates of yeast and lactic acid bacteria were provided by Dr. S. Blasche (EMBL, Germany). Wine yeast strains were provided by Dr. R. González (Instituto de Ciencias de la Vid y del Vino, Logroño, Spain).

Chemically defined medium (CDM35) was designed by reducing the number of components in the rich medium composed as a union of previously described media (Verduyn et al., 1992, Wegkamp et al., 2010, Zhang et al., 2009) (Table S1). Rapamycin (Sigma-Aldrich) concentration was 20 nM, unless stated otherwise. Addition of the rapamycin directly to the LAB cultures did not affect their growth (Figure S13). Lactose concentration in the medium was 0.5%. Organic grape juice was purchased in a local store and filter-sterilized before using in co-culture experiments.

Liquid medium experiments were started at 0.01 OD_600_ using pre-cultures washed 2 times with phosphate buffered saline (PBS). All cultures were grown statically at 30°C. Liquid CDM35-Lactose cultures were incubated for 7 days.

Solid medium cultures were started by spotting 5 μl of the washed LAB cultures (0.1 OD_600_) and yeast culture (0.2 OD_600_) on CDM35 plates (2% agar). LAB cultures were placed in proximity to the yeast inoculum and further away on the same plate as a control. Plates were imaged after 2-3 days of incubation at 30°C. CDM35-Lactose agar plates were incubated for 9 days.

To measure growth rate of yeast knockout strains in CDM35, cultures were grown statically in 96 well microtiter plates, OD_600_ measurements were made every 15 min using BioTek Synergy microplate reader.

#### Yeast Strains Construction

Four knockout mutants (*ure2Δ*, *gtr1Δ*, *dal81Δ*, *gln3Δ*) were constructed in the S90 genetic background. Genomic DNA of four mutants in BY4741 background (Mülleder et al., 2012) was extracted and fragments containing kanMX4 cassette with 200-400 base pairs of flanking genomic regions were PCR amplified using previously described A-D primer pairs (Winzeler et al., 1999). These PCR products were used to transform wild type *S. cerevisiae* S90 as described previously (Gietz and Schiestl, 2007) with some modifications. Yeast mid-log culture (OD_600_ = 0.7, 50 ml) was centrifuged, washed, and re-suspended in 1 ml of sterile water. Then 100 μl of cell suspension was topped with a transformation mix (240 μl PEG 3500, 50% w/v; 36 μl lithium acetate, 1.0 M; 50 μl boiled single-stranded carrier DNA, 2 mg/ml; 34 μL of PCR amplification product described above) and re-suspended. After incubation for 40 min (42°C water bath), cells were re-suspended in YPAD and incubated for 3-4 hours to allow for the expression of the integrated antibiotic marker. Clones were selected on YPAD medium with G418 antibiotic (300 μg/ml). Success of homologous recombination was verified by colony PCR using A-D, A-KanB, and C-KanD primer pairs (Table S6) as described in (Winzeler et al., 1999).

#### Conditioned Medium Assay

Yeast culture (∼1 OD_600_) was centrifuged, supernatant passed through 0.2 μm syringe filter and used to culture LAB species for 24 hours. To test yeast strains with TORC1-related gene deletions 82 strains were picked from the prototrophic single gene knockout library (Mülleder et al., 2012).

### Method Details

#### Quantification of Species in Communities

##### Counting Colony Forming Units

For co-culture stability testing, all combinations of three species were passaged by transferring 20 μl of culture into 2 ml of fresh CDM35 medium every 24 hours. CFUs were counted by plating each of 3 biological replicates in 3 technical replicates onto selective media. MRS and GM-17 agar plates supplemented with 10 μg/ml cycloheximide were used to selectively estimate quantities of *L. plantarum* and *L. lactis* respectively, and Synthetic Defined (SD) medium was used to select for yeast. The concentration of cycloheximide used was verified to have no effect on LAB. Plates were incubated for 2 days at 30°C before counting.

##### Species-Specific qPCR

Species-specific primers were designed (Table S6) and tested against other species in the community. Templates for qPCR analysis were prepared as described below. In brief, 1 ml of (co-)culture was centrifuged, and pelleted cells flash-frozen and stored until analysis. Frozen samples were re-suspended in 1 ml of deionized water, 400 μl of mixture transferred into a polypropylene screw cap tube containing 0.5 ml of acid washed glass beads (0.2-0.3 mm). Homogenization was done in a FastPrep-24 bead beater (4 m/s setting, for 2 min with intermittent cooling on ice). Lysate was diluted 1000 times with an alkaline PEG reagent (60% PEG 200, 20 mM potassium hydroxide, pH 13.3-13.5 as described in (Chomczynski and Rymaszewski, 2006) and incubated for 10 min at 95°C. 1 μl of resulting sample was used directly for qPCR reaction.

qPCR reaction mix was prepared using 1 μl template and 19 μl of SYBR Green RT-PCR master mix (Life Technologies), 0.5 μM primers (synthesized and desalted by Sigma-Aldrich). Amplification was performed on StepOne Plus real-time PCR system (Applied Biosystems), 40 cycles, 60°C annealing temperature. Quantification was done against serial dilutions of corresponding monocultures of a known optical density, with a standard curve generated on every microtiter plate. Data analysis was done using StepOne software (Applied Biosystems).

##### Flow Cytometry

0.5 ml of *L. lactis* (kefir) (co-)culture was pelleted, washed with 70% ethanol, then rehydrated in phosphate buffered saline with 1 mM EDTA to a final density of 0.6 OD_600_. Propidium iodide (PI, Sigma-Aldrich) was used as a fluorescent probe to label bacterial DNA. PI was added in EDTA buffer to 3 μg/mL final concentration, vortexed and incubated for 10 min in darkness. Each sample was then sonicated 5 times (10 seconds with 0.5 second ON-OFF intervals; 10% amplitude; Branson Sonifier W-250 D, Heinemann) at 4°C, interrupted by cooling on ice. Absolute bacterial cell numbers were determined by addition of 10 μl of CountBright™ absolute counting beads (Thermo Fisher). PI and the fluorescence beads were simultaneously analyzed by flow cytometry using FACS BD Accuri™ C6 Cytometer (BD Biosciences) at low flow. PI and beads were monitored in channels FL2 and FL4 respectively. A total of 10,000 PI positive cells were analyzed for each sample, thresholds used were 800 FSC-H and 600 FL2-H. Yeast cells were excluded from counts of the mixed cultures based on size and fluorescence.

#### Assessment of Yeast Cell Death/Damage

LIVE/DEAD FungaLight Yeast Viability Kit (Life Technologies) was used in compliance with manufacturer instructions to access fraction of dead yeast cells and/or cells with compromised membranes in five strains of S90 background (*Δure2*, *Δgtr1*, *Δdal81*, *Δgln3* and the wild type). Exponentially growing cells were washed and re-suspended in Tris buffered saline solution, pH 7 to produce a suspension of 0.4 OD_600_. Cells were stained with PI and SYTO® 9 fluorescent dyes and counted with LSR-Fortessa analyzer (Beckton Dickinson). Fluorescent events were recorded at excitation/emission 480/500 nm for SYTO9 and 490/635 nm for PI. Gating was adjusted to exclude debris and cell duplicates. Each sample was measured until the minimum of 2000 of dead/damaged cells was reached. See Figures S7B and S7C for the results.

#### Untargeted Exo-Metabolome Analysis

Samples of supernatant were taken at multiple time-points during the conditioning of the medium with yeast (until 18-hour time point when conditioned medium was harvested) and then during the growth of individual *L. lactis* and *L. plantarum* cultures. Collectrd samples were passed through 0.2 μm PVDF syringe filter and 3-kDa MWCO centrifugal filters (Millipore). Ten-fold dilutions were analyzed on a platform consisting of an Agilent 6550 ion funnel QTOF mass spectrometer coupled to a Gerstel MPS2 autosampler and a Hitachi L-7100 HPLC pump operated using published settings (Fuhrer et al., 2011). The isocratic flow rate was 150 μl/min of mobile phase consisting of isopropanol:water (60:40, v/v) buffered with 5 mM ammonium fluoride at pH 9 for negative ionization mode. For online mass axis correction, 2-propanol (in the mobile phase), taurocholic acid and Hexakis (1H, 1H, 3H-tetrafluoropropoxy)phosphazine were used. Mass spectra were recorded in profile mode from 50 to 1000 m/z with a frequency of 1.4 spectra/sec using the highest available resolving power (4 GHz HiRes). Source temperature was set to 325°C, with 5 l/min drying gas and a nebulizer pressure of 30 psi. Fragmentor, skimmer, and octupole voltages were set to 175 V, 65 V and 750 V respectively. Ions were annotated as metabolites within 0.005 Da tolerance using the KEGG database as reference considering [M-H]^-^ and [M+F]^-^ as dominant ions as well as H/K and H/Na exchange and NaCl adducts as possible neutral gains.

#### RNA Extraction, Sequencing and Data Analysis

Yeast cultures were harvested at OD_600_ ∼1 by mixing with an equal volume of cold methanol, centrifuging at -9°C, and storing pellets at -80°C. Total RNA was extracted using RNeasy Mini Kit (QIAGEN) according to the manufacturer’s instructions. Customized steps included homogenization with glass beads, as described for qPCR procedure, and on-column DNA digestion step using Turbo DNAse (Invitrogen). RNA was quantified with Qubit 2.0 fluorometer (Invitrogen) using the Qubit RNA broad range assay kit. cDNA library was prepared from starting amount of 500 ng total RNA according to Illumina’s RNA-Seq protocol (http://www.illumina.com/products/tru seq_rna_sample_prep_kit_v2.ilmn) using Beckman Biomek FX laboratory automation workstation. Prepared library samples were multiplexed 12 samples per lane and sequenced with HiSeq2000 (Illumina) platform, generating 50 base pair long unpaired end reads.

The quality of the RNA sequencing reads was assessed with FastQC (https://www.bioinformatics.babraham.ac.uk/projects/fastqc/). End trimming and filtering for fragments over 30 base pair long with quality score over 30 was performed using FaQCs (Lo and Chain, 2014). The reads were then mapped to the *S. cerevisiae* S288c reference genome (R64-1-1) using TopHat (Trapnell et al., 2009). Read counts for all genes were extracted with HTSeq-count (Anders et al., 2015) and normalized using the R package DESeq2 (Anders and Huber, 2010). For further statistical analysis the count data were filtered for average read count above 10 and transformed with the regularized logarithm transformation (rlog) of DESeq2.

#### Metabolic Modeling of Community Cross-Feeding

Automatically reconstructed genome-scale metabolic models and manually curated *L. lactis* IL1403 model (Oliveira et al., 2005) were obtained from the ModelSEED (Henry et al., 2010) database. Manually curated models of *L. plantarum* and *S. cerevisiae* were obtained from (Teusink et al., 2006, Zomorrodi and Maranas, 2010). Manually curated model of *Lactococcus lactis* IL1403 was updated in order to reconcile model’s *in silico* growth with *L. lactis* IL1403 inability to growth in the CDM35 medium and its known auxotrophies (Table S4). In summary, uptake reactions for all amino acids and 2-oxoglutarate were added, as well as two new reactions that enable biosynthesis of proline and lysine, similarly to *L. lactis* MG1363 metabolic model (Flahaut et al., 2013). Furthermore, biosynthesis of leucine, arginine, valine, glutamine, glutamate and isoleucine was blocked, as *L. lactis* IL1403 is known to be unable to synthesize these metabolites (Table S4).

To simulate metabolic exchanges, individual species models were combined into a community model. Hereby each species would interact with a common external metabolic environment through their metabolite exchange reactions. This allowed every member species access to the pool of media metabolites and metabolites secreted by other species. Each species could secrete/uptake only those metabolites for which an exchange reaction (e.g. via transporters or free diffusion) exists in the model. Metabolite exchanges in the community were simulated using SMETANA framework as introduced in (Zelezniak et al., 2015). In brief, the growth of each community member was imposed as a constraint, and subsequently the space of all possible metabolic exchanges in CDM35 medium was systematically enumerated by solving a series of mixed-integer linear programming problems. No assumptions of growth optimality were made at the level of individual species or the community.

#### Amino Acid Quantification (LC-MS)

Yeast conditioned medium was passed through 0.2 μm PVDF syringe filters and 3 kDa MWCO centrifugal filters (Millipore), diluted 1:10, then 1 μl was used for amino acid analysis on a liquid chromatography (Agilent 1290 Infinity) and tandem mass spectrometry (Agilent 6460) system, as described elsewhere (Mülleder et al., 2016a). Method covered all proteinogenic amino acids (except cysteine), ornithine, citrulline, and α /γ-aminobutyric acid. In short, amino acids were separated by hydrophilic interaction chromatography with gradient elution on a Waters ACQUITY UPLC BEH Amide column (2.1 x 100 mm, 1.7 μm) using a binary solvent system of 50:50 acetonitrile:water and 95:5:5 acetonitrile:methanol:water, both containing 0.176% formic acid, 10 mM ammonium formate. The compounds were identified by comparing retention time and fragmentation pattern with analytical standards (Sigma-Aldrich). The obtained signals, operating the instrument in selected reaction monitoring mode, were processed and quantified by external calibration with Agilent MassHunter software.

#### Amino Acid and Lactose Quantification (GC-MS)

Supernatants of yeast monoculture and yeast-*L. lactis* co-cultures in CDM35-lactose were filtred with 0.2 μm PVDF syringe filter and 3kDa MWCO filter (Millipore). Ribitol (Adonitol) (Alfa Aesar, UK) was added as an internal standard. Polar metabolites were extracted from 100 ul of the sample by addition of 200 μL of methanol (Biosolve Chimie, France), followed by incubation at 72°C for 15 min and addition of 200 μL of MilliQ water. The dried extracts were derivatized to (MeOx)TMS-derivatives through reaction with 100 μL of 20 mg/mL methoxyamine hydrochloride (Alfa Aesar, UK) solution in pyridine (Alfa Aesar, UK) for 90 min at 40°C, followed by reaction with 200 μL N-methyl-trimethylsilyl-trifluoroacetamide (MSTFA) (Alfa Aesar, UK) for 12 hours at room temperature, as described previously (Kanani et al., 2008). Metabolites were measured using a Shimadzu TQ8040 triple quadrupole GC-MS system. The gas chromatograph was equipped with a 30 m x 0.25 mm x 0.25 μm DB-50MS capillary column (Phenomenex, USA). The detector was operated both in scan mode recording in the range of 50-600 m/z, as well as in MRM mode to monitor amino acids and lactose.

#### Amino Acid and Polyamines Detection (UPLC)

Additional amino acids and polyamines quantification in yeast conditioned medium was done by fluorescent labeling using AccQ-TagTM (Waters) according to the manufacturer’s protocol. The resulting derivatives were on Acquity BEH C18 column (150 mm x 2.1 mm, 1.7 μm, Waters) connected to an Acquity H-class UPLC system and quantified by fluorescence detection (Acquity FLR detector, Waters, excitation: 250 nm, emission: 395 nm) using ultrapure standards (Sigma-Aldrich). The column was heated to 42°C and equilibrated with 5 column volumes of buffer A (140 mM sodium acetate pH 6.3, 7 mM triethanolamine) at a flow rate of 0.45 ml per minute. Gradual increase of acetonitrile (B) in buffer A was set up as follows: 1 min 8% B, 16 min 18% B, 23 min 40% B, 26.3 min 80% B, hold for 5 min, and return to 8% B in 3 min. Data acquisition and processing were performed with the Empower3 software suite (Waters).

#### Measurement of Branched-Chain α-Ketoacids

To analyze the content of branched-chain α-ketoacids, 100 μl of yeast conditioned medium was mixed with 200 μl cold 1M perchloric acid. Insoluble materials were removed by centrifugation, 150 μl of the resulting supernatant were mixed with an equal volume of 25 mM o-phenylendiamine solution and derivatized by incubation at 50°C for 30 min. After centrifugation the derivatized keto-acids were separated by reversed phase chromatography on an Acquity HSS T3 column (100 mm x 2.1 mm, 1.7 μm, Waters) connected to an Acquity H-class UPLC system. Prior to separation, the column was heated to 40°C and equilibrated with 5 column volumes of solvent A (0.1% formic acid in 10% acetonitrile) at a flow rate of 0.55 ml/min. Separation of ketoacid derivates was achieved by increasing the concentration of solvent B (acetonitrile) in solvent A as follows: 2 min 2% B, 5 min 18% B, 5.2 min 22% B, 9 min 40% B, 9.1min 80% B and hold for 2 min, then return to 2% B in 2 min. The separated derivatives were detected by fluorescence (Acquity FLR detector, Waters, excitation: 350 nm, emission: 410 nm). Data acquisition and processing were performed with the Empower3 software suite (Waters).

### Quantification and Statistical Analysis

#### Estimation of Amino Acid Leakage

Parameters: Cell volume = 45.54 fL; Number of cells per mL per OD = 32 million. Intra-cellular amino acid concentrations are taken from (Mülleder et al., 2016b). Example calculation for alanine: intra-cellular concentration is 6 mM. Estimated release of alanine for death rate of 0.04% (average for wild type cells, see Figure S7) = 3.5 10^-06^ mM, which is ∼20000 times less than concentration observed in the conditioned medium. Even for the highest death rate estimate across all tested mutants (0.22%), the observed concentration is several thousand fold more than that could be explained by cell lysis.

#### Statistical Analysis

For comparing conditioned media prepared with different strains/media, yeast cultures were harvested at similar OD_600_ (∼1) and resulting values of LAB growth were normalized by the OD_600_ of the corresponding yeast cultures. For amino acid quantification samples with OD_600_ over 1.5 were excluded from analysis. Screening was done once in two technical replicates, and repeated at least twice more for the strains that showed differential effect on LAB growth. Growth rate (μ max) was estimated as a maximum slope of fitted model-free smoothed spline using “grofit” R package (Kahm et al., 2010). P-values for comparisons between conditions were estimated using an unpaired two-tailed t-test. Statistical details of the experiments can be found in the figure legends.

Identification of gene sets with expression positively associated with growth enabling effect of yeast on LAB was done as follows. First, for each gene we calculated coefficients of correlation (Spearman and Pearson) between its expression values in yeast deletion strains and bacterial final OD_600_ in the conditioned media of corresponding yeast strains. Ranking gene list by both correlation coefficients allowed generation of z-scores that were transformed into p-values and used as an input for calculating gene set enrichment statistics using “piano” R package (Varemo et al., 2013). “Reporter” method was used, with P-values calculated from a theoretical null distribution (10,000 permutations). P-values were corrected for multiple testing using Benjamini-Hochberg procedure. Pathway enrichment was calculated for a gene sets (with at least 5 genes in each group) composed of KEGG pathways, biochemical pathways from the Saccharomyces Genome Database (SGD), targets of transcription factors *DAL81* and *GLN3* according to Yeastract database (Teixeira et al., 2014) (DNA binding and expression evidence), and gene sets of NCR pathways and amino acid transporters as defined by (Ljungdahl and Daignan-Fornier, 2012).

### Data and Software Availability

Transcriptome dataset for five yeast strains of S90 genetic background (*ure2Δ*, *gtr1Δ*, *dal81Δ*, *gln3Δ* and the wild type) was deposited in the http://www.ebi.ac.uk/arrayexpress/ under accession number E-MTAB-4651. An updated *L. lactis* model, iAOP358, is available at https://www.patil.embl.de/media/models/ponomarova/iAOP358.bioopt. metabolomics datasets are available at http://dx.doi.org/10.17632/r5bx5cg9y6.1 (FIA-TOF MS data) and http://dx.doi.org/10.17632/h2fvn2mpp3.1 (LC-MS/MS Metabolomics data).

## Author Contributions

O.P. conceived the project, performed experiments and modeling, analyzed the data, and wrote the paper. N.G. performed nitrogen load, grape juice, and CDM35-lactose experiments. D.S. performed the exo-metabolome mass spectrometry analysis. M.M. and M.R. provided support for targeted metabolomics. E.K. performed the GC-MS analysis. K.Z. analyzed RNA-seq data. K.B. helped with the media design experiments. S.A. contributed to modeling and data analysis. A.T. contributed to experimental design. U.S. oversaw the exo-metabolome analysis. M.R. oversaw the targeted metabolomics analysis. K.R.P. conceived the project, oversaw the project, and wrote the paper.

## Supplemental Information

Document S1. Figures S1–S13 and Tables S1 and S3–S8

Table S2. Ion Annotation for Candidate Exchange Metabolites Identified by Untargeted Metabolomics, Related to Figure 2

Document S2. Article plus Supplemental Information
