# Supplementary material for: Yeast Creates a Niche for Symbiotic Lactic Acid Bacteria through Nitrogen Overflow
Source: Cell Syst. 2017 Oct 25;5(4):345–357.e6. doi: 10.1016/j.cels.2017.09.002 (PMC5660601; doi:10.1016/j.cels.2017.09.002)
Supplement: Document S1. Figures S1–S13 and Tables S1 and S3–S8 [file mmc1.pdf]

Cell Systems, Volume *5*

Supplemental Information

Yeast Creates a Niche for Symbiotic Lactic Acid Bacteria through Nitrogen Overflow

Olga Ponomarova, Natalia Gabrielli, Daniel C. Sévin, Michael Mülleder, Katharina Zirngibl, Katsiaryna Bulyha, Sergej Andrejev, Eleni Kafkia, Athanasios Typas, Uwe Sauer, Markus Ralser, and Kiran Raosaheb Patil

Supplemental Tables

Table S1. Related to STAR methods (Strains, media and growth conditions). CDM35 medium composition (final pH 7).

	Component	Concentration, g/L
**Carbon Source**	**Glucose**	15
**Amino Acids**	**L-Arginine**	0.72
	**L-Asparagine**	0.5
	**L-Histidine**	0.17
	**L-Isoleucine**	0.24
	**L-Leucine**	1
	**L-Methionine**	0.125
	**L-Tyrosine**	0.3
	**L-Valine**	0.7
**Vitamins**	**Biotin**	0.006
	**Ca pantothenate**	0.0012
	**Folic acid**	0.00056
	**myo-Inositol**	0.002
	**Nicotinic acid**	0.0009
	**p-Aminobenzoic acid**	0.000056
	**Pyridoxine HCl**	0.0048
	**Riboflavin**	0.0009
	**Thiamine HCl**	0.00056
**Inorganic Salts**	$FeSO_4 \cdot 7H_2O$	0.005
	K_2HPO_4	6.48
	KH_2PO_4	3.12
	$MgCl_2$	0.3864
	**NaCl**	3
	$ZnSO_4$	0.005
	K_2SO_4	0.023
	**Boric acid**	0.00075
	$CaCl_2$	0.03
	$CoCl_2 \cdot 6H_2O$	0.00019
	$CuSO_4$	0.00012
	**KI**	0.00011
	$MnSO_4 \cdot H_2O$	0.00034
	$(NH_4)6Mo_7O_{24} \cdot 4H_2O$	0.00019

**Other**	**Ammonium citrate dibasic**	1.69
	**Citric acid·H_2O**	0.003
	**L-Glutathione reduced**	0.015

Table S2. Related to Figure 2. Ion annotation for candidate exchange metabolites identified by untargeted metabolomics.

Included separately as Excel file.

Table S3. Related to Figure 3. Metabolites detected in yeast conditioned medium in addition to amino acids.

Metabolite	Concentration, μM
Putrescine	71
Phenylpyruvate	0.92
Pyruvate	149
2-Oxoglutarate	30
α-Ketoisocaproic acid	4

Table S4. Related to the Figure 3, Figure S4, and STAR Methods (Metabolic modeling of community cross-feeding). Amino acid requirements of *Lactococcus lactis* IL1403 and *Lactobacillus plantarum* WCFS1.

E-essential; S-stimulatory amino acids

Amino acid	Reference				
	L. lactis IL1403				*L. plantarum* WCFS1
	(Van Niel and Hahn-Hägerdal, 1999)	(Zhang et al., 2009)	(Cocaign-Bousquet et al., 1995)	(Aller et al., 2014)	(Wegkamp et al., 2010)
Alanine		S		S	
Arginine	E	E	E	E	E
Asparagine	E	S		E	
Cysteine	S			S	S
Glutamine	E			S	
Glutamate			E		E
Glycine		S			
Histidine	E	E	E	E	
Isoleucine	E	E	E	E	E
Leucine	E	E	E	E	E
Lysine		S		S	
Methionine	E	E	E	E	E
Phenylalanine					E
Proline		S			
Serine	S	S	E	E	
Threonine	S	S	E	S	E
Tryptophan					E
Tyrosine		S			E
Valine	E	E	E	E	E

Table S5. Related to the Figure 4. Effect of yeast strains with deletions of TORC1-related genes on LAB growth in CDM35 conditioned medium (relative to the wild type).

Note: Deletion of *GLN3* reduces the growth of *L. lactis* but not *L. plantarum*.

neutral		non-viable	increase	decrease
AGP1	*PUT2*	*ARG4*	*ALT1*	*DAL81*
ARG4	*SFA1*	*ATG5*	*EGO3*	*GLN3*
ARO10	*SIT4*	*AVT1*	*GCN1*	
ARO80	*STP1*	*AVT4*	*GTR1*	
ARO9	*STP2*	*BAP3*	*LST4*	
ASN1	*TOR1*	*BAT2*	*PEP3*	
ASP1	*UGA1*	*CAN1*	*URE2*	
ATG1	*UGA2*	*DAL82*		
AVT6	*UGA3*	*DIP5*		
CAR1	*VID30*	*DUR3*		
CAR2		*GRR1*		
DAL1		*GUD1*		
DAL2		*GZF3*		
DAL3		*HOM2*		
DAL4		*HOM3*		
DAL5		*MKS1*		
DAL7		*NCR1*		
DAL80		*NCR2*		
DUR1,2		*NPR1*		
FPR1		*NPR2*		
GAP1		*PDR12*		
GAT1		*RTG1*		
GCN2		*RTG3*		
GCN4		*SCH9*		
GLT1		*TAT1*		
GNP1		*TAT2*		
LEU3		*TOD6*		
MEP3		*UGA4*		
MSN2		*VPS52*		
MSN4		*YCK1*		
PTR2		*YCK2*		
PUT1				

Table S6. Related to STAR Methods (Quantification of species in communities). Species-specific primers used for quantification of the microorganisms in communities.

Primer sequence		Usage	Target species	Target gene	Amplicon size	Reference
**Forward (5'-3')**	**Reverse (5'-3')**					
AGTGGCCTACCATGGTTTCA	CTTGGATGTGGTAGCCGTTT	qPCR	*Saccharomyces cerevisiae*	18S rRNA	86	This paper
GCAGGCGTAACTAAAGCAGC	AAGCGTTTCAGCAGGGGTAA	qPCR	*Lactobacillus plantarum*	HAD (HAD superfamily hydrolase)	72	This paper
GGCGCTCTAAATCGAGTCGA	GCAAAGCCTGACTTGCTGTC	qPCR	*Lactococcus lactis*	dnaA	82	This paper
AATACCGACTGTTGTCTGGAATAAC	TCATAACTGAACAAATCCCTTCTTC	knockout construction	*Saccharomyces cerevisiae*	YNL229C	N/A	(Winzeler et al., 1999)
CAGAGTATACCGAGTCGTTTGAAGT	AACAAAACCTAACACACACACACAC	knockout construction	*Saccharomyces cerevisiae*	YML121W	N/A	(Winzeler et al., 1999)
TCCCACAATAACAGAGTGTGTAAGA	AAACAAATAATACCAATGCTCAGGA	knockout construction	*Saccharomyces cerevisiae*	YER040W	N/A	(Winzeler et al., 1999)
TCAACAGAGATGATTTGTGTCATTT	GCAAAGTATAATAGACGAGGCAAAA	knockout construction	*Saccharomyces cerevisiae*	YIR023W	N/A	(Winzeler et al., 1999)
TGATTTTGATGACGAGCGTAAT	CTGCAGCGAGGAGCCGTAAT	knockout verification	N/A	KanMX4 module	N/A	(Winzeler et al., 1999)

Table S7. Related to Figure 5B. Amino acid content of the media used for the varying nitrogen load experiment, as measured by targeted LC-MS. Also included is the yeast growth in the corresponding media.

Amino acid, μM	Relative N load					
	1.00	0.17	0.08	0.04	0.02	0.01
Phenylalanine	0.00	0.00	0.00	0.00	0.00	0.00
Leucine	39854.92	7490.42	3860.28	2171.90	1171.62	733.34
Isoleucine	1645.32	292.14	183.84	93.87	52.50	30.38
Methionine	643.12	56.20	15.41	6.20	3.51	1.85
Valine	7154.85	1183.81	593.90	297.67	143.32	86.24
Proline	0.00	0.00	0.00	0.00	0.00	0.00
Tyrosine	3956.07	1255.20	723.40	480.34	301.98	240.36
Alanine	0.00	0.00	0.00	0.00	0.00	0.00
Threonine	0.00	0.00	0.00	0.00	0.00	0.00
Homoserine	0.00	0.00	0.00	0.00	0.00	0.00
Glycine	0.00	0.00	0.00	0.00	0.00	0.00
Glutamine	0.00	0.00	0.00	0.00	0.00	0.00
Glutamate	0.00	0.00	0.00	0.00	0.00	0.00
Serine	0.00	0.00	0.00	0.00	0.00	0.00
Asparagine	2473.64	615.89	366.42	197.80	122.31	80.89
Citrulline	0.00	0.00	0.00	0.00	0.00	0.00
Aspartate	0.00	0.00	0.00	0.00	0.00	0.00
Arginine	7030.94	1281.18	310.52	225.26	163.28	134.94
Histidine	5823.65	2207.85	754.09	511.29	347.56	240.26
Tryptophan	0.00	0.00	0.00	0.00	0.00	0.00
Lysine	0.00	0.00	0.00	0.00	0.00	0.00
Yeast Growth (OD_{600})	1.33±0.08	1.36±0.11	1.42±0.05	1.16±0.09	1.33±0.18	1.25±0.04

Table S8. Related to Figure 5C. Amino acid content of 3 different lots of grape juice measured by targeted LC-MS.

Amino acid (mM)	Grape Juice		
	Lot nr. 08114	Lot nr. 13523	Lot nr. 09454
Phenylalanine	5.78	5.67	5.99
Leucine	7.67	7.68	0.80
Isoleucine	1.21	1.20	1.26
GABA	0.00	0.00	0.00
Methionine	4.76	3.59	5.01
Valine	2.38	2.39	2.52
Proline	1.95	2.06	2.22
Tyrosine	2.64	2.71	2.87
AABA	0.00	0.00	0.00
Alanine	1.25	1.29	1.38
Threonine	0.47	4.80	5.18
Homo-serine	0.00	0.00	0.00
Glycine	1.14	1.14	1.28
Glutamine	3.70	2.57	1.89
Glutamate	4.68	5.11	5.15
Serine	3.40	3.44	3.55
Asparagine	8.80	9.74	1.10
Citrulline	0.00	0.00	0.00
Aspartate	9.59	9.45	1.06
Arginine	2.82	3.38	3.88
Histidine	0.73	7.84	7.45
Tryptophan	9.59	0.22	6.77
Lysine	9.41	9.74	1.03

Supplemental Figures

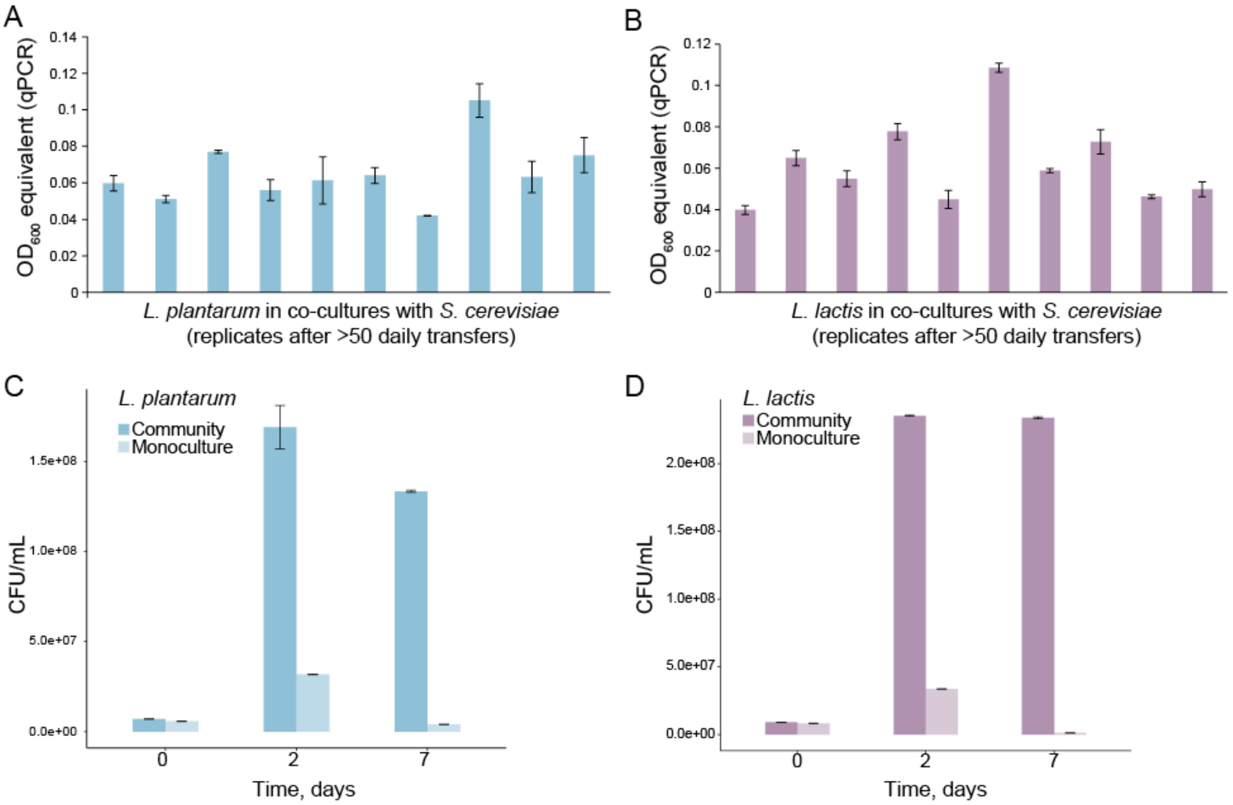

Figure S1. Related to Figure 1. (A, B) Lactic acid bacteria remain in co-culture with *S. cerevisiae* for over fifty days of daily passaging. Error bars, mean ± s.d., (n = 3 technical replicates). Each bar represents a biological replicate. (C, D). Lactic acid bacteria survive in a week-long co-cultivation (without sub-culturing) with yeast but not in monocultures. Error bars, mean ± s.d., (n = 3 independent biological replicates) in all graphs.

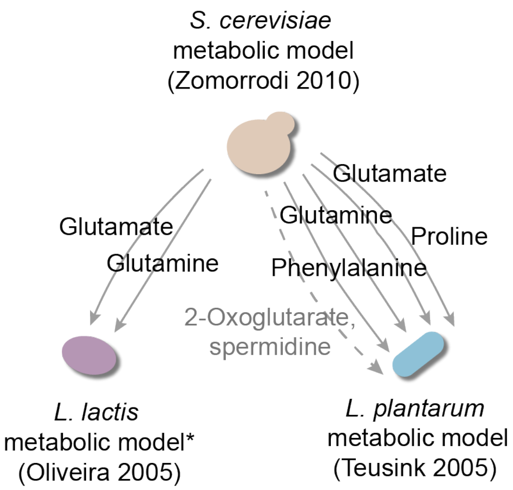

Figure S2. Related to STAR Methods. Predictions of exchanged metabolites based on genome-scale metabolic modeling. Manually curated models of the individual species were used to reconstruct community models (see STAR Methods). Dashed line denotes predictions when using automatically reconstructed model for *L. plantarum*. * indicates updated model.

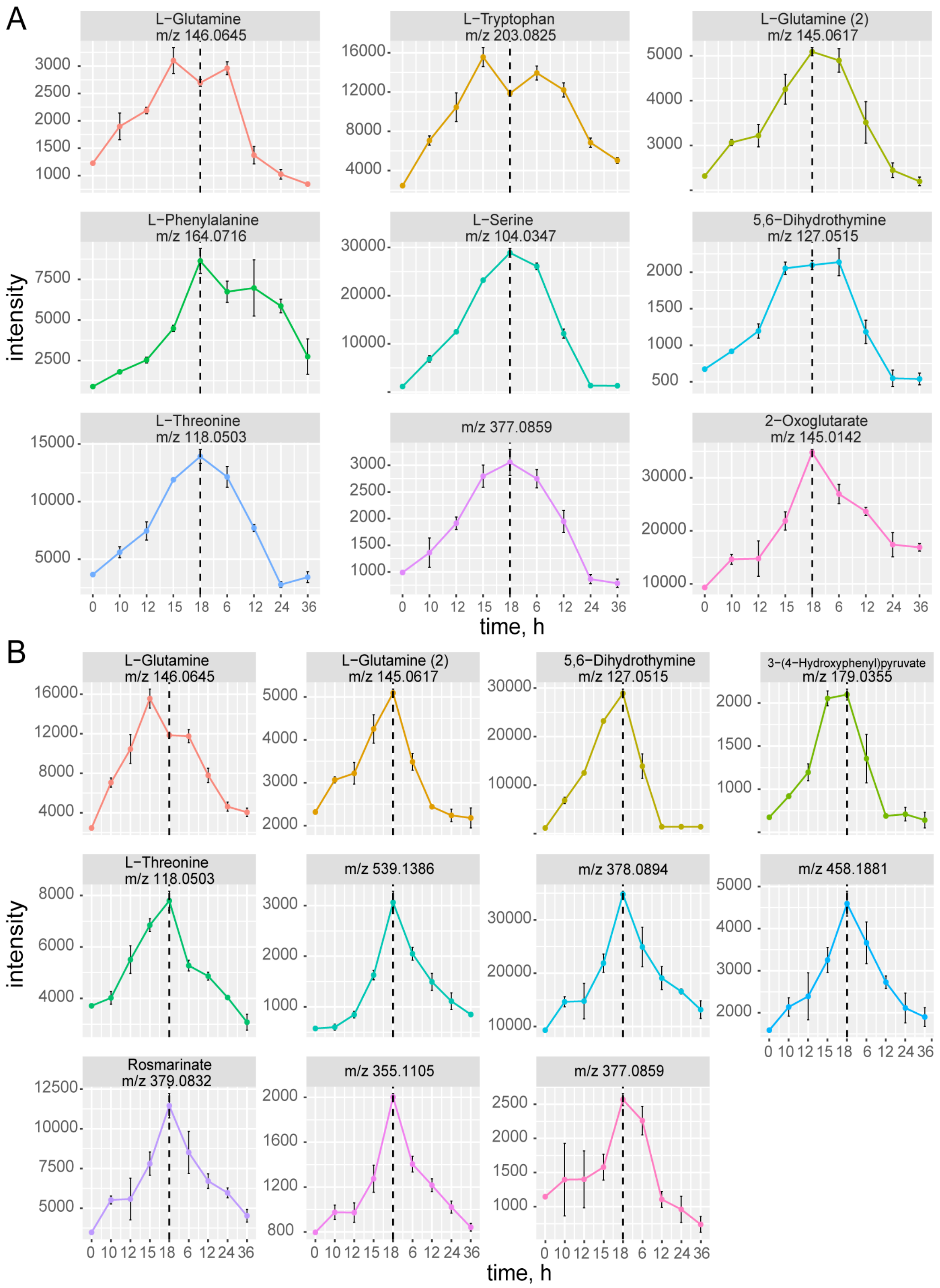

Figure S3. Related to Figure 2. Metabolites produced by *S. cerevisiae* and consumed by lactic acid bacteria (untargeted metabolomics). (A and B) Metabolites consumed by *L. plantarum* and *L. lactis,* respectively. Metabolite dynamics in yeast and LAB cultures are separated by dashed line. Shown are ions with at least two-fold change in both accumulation and decrease. Error bars, mean ± s.d., (n = 3 independent biological replicates).

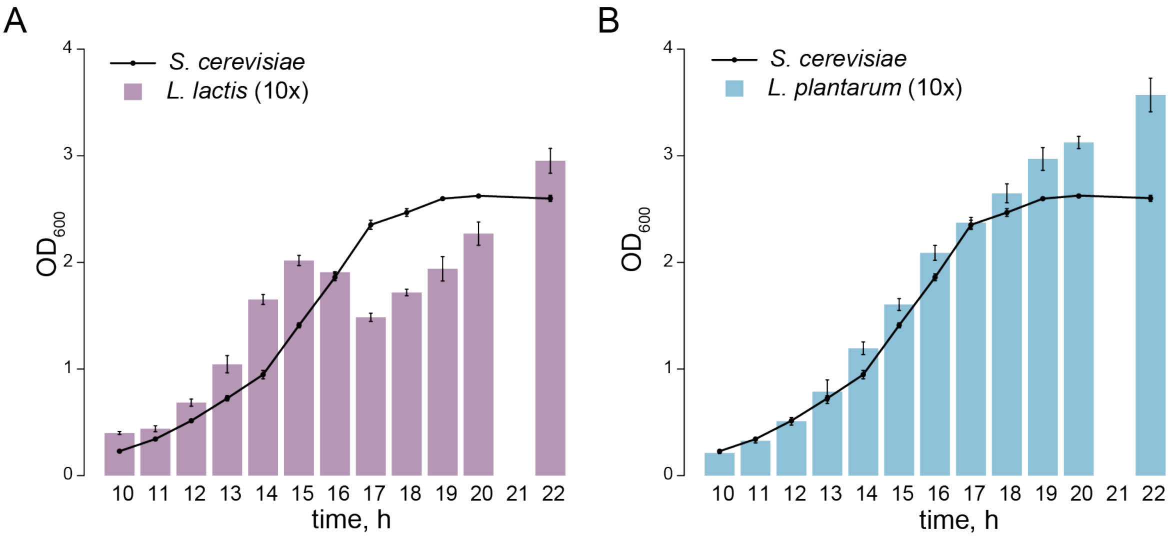

Figure S4. Related to Figure 3. Effect of yeast conditioned medium collected at different yeast growth stages on LAB growth. (A and B) Black line shows OD_{600} of corresponding yeast culture. Error bars, mean ± s.d., (n = 4 technical replicates).

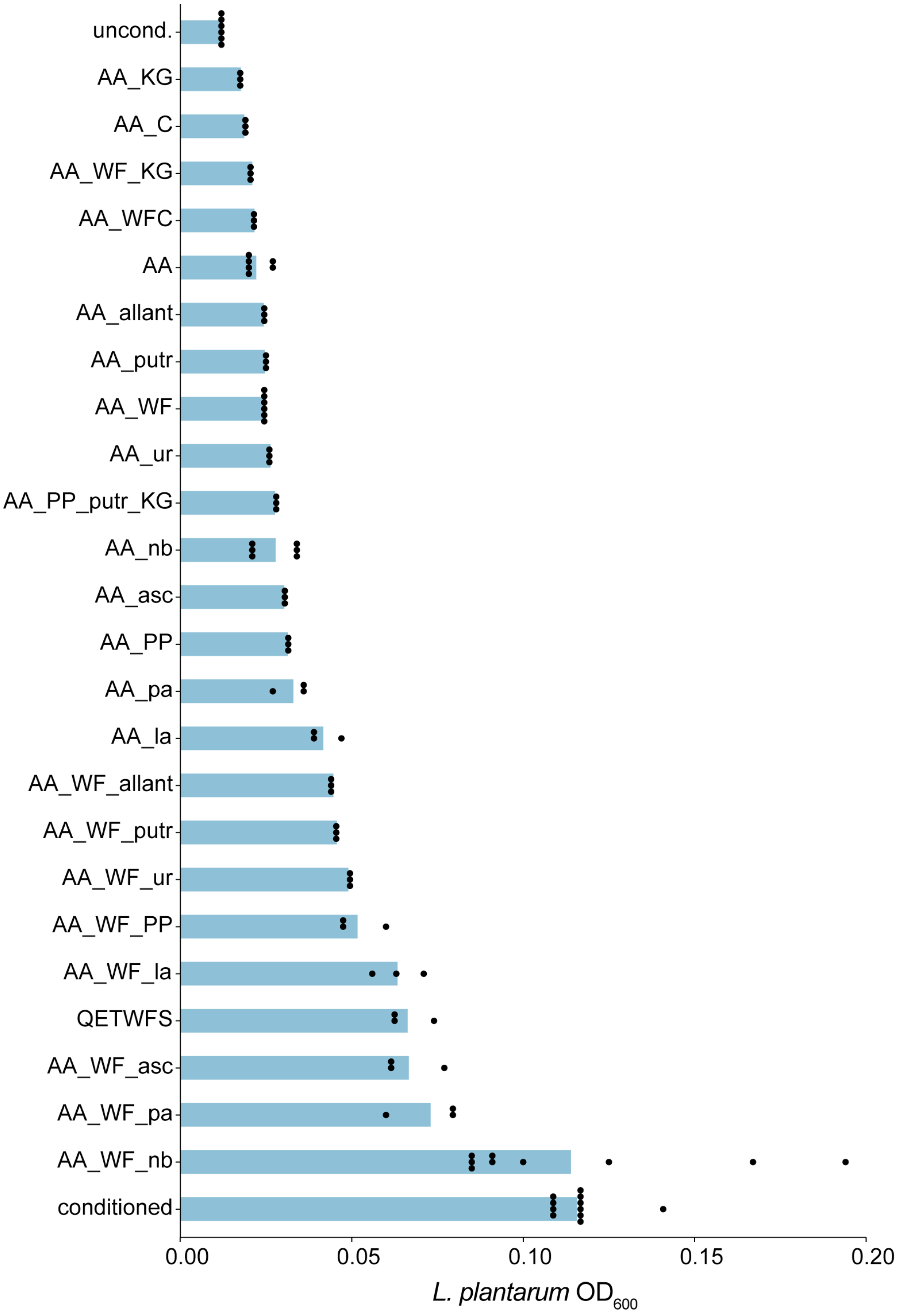

Figure S5. Related to Figure 3. *L. plantarum* growth in supplemented CDM35 medium. CDM35 was supplemented with: AA – seven amino acids identified in the yeast conditioned medium (in respective concentrations), KG – alpha-ketoglutarate (200 µM), allant – allantoin (200 µM) , putr – putrescine (200 µM), ur – urea (250 µM), PP – sodium phenylpyruvate and hydroxy phenylpyruvate (200 µM each) , W- tryptophan (1 mM), F – phenylalanine (2.4 mM), C – cysteine (1.6 mM), la – lipoic acid (6 µM), asc – ascorbate (2.8 mM), nb- nucleobases adenine (80 µM), guanine (36 µM), uracil (200 µM), and xanthine (24 µM), pa – pyridoxamine dihydrochloride (30 µM). Dots represent pooled technical replicates of at least two independent biological replicates.

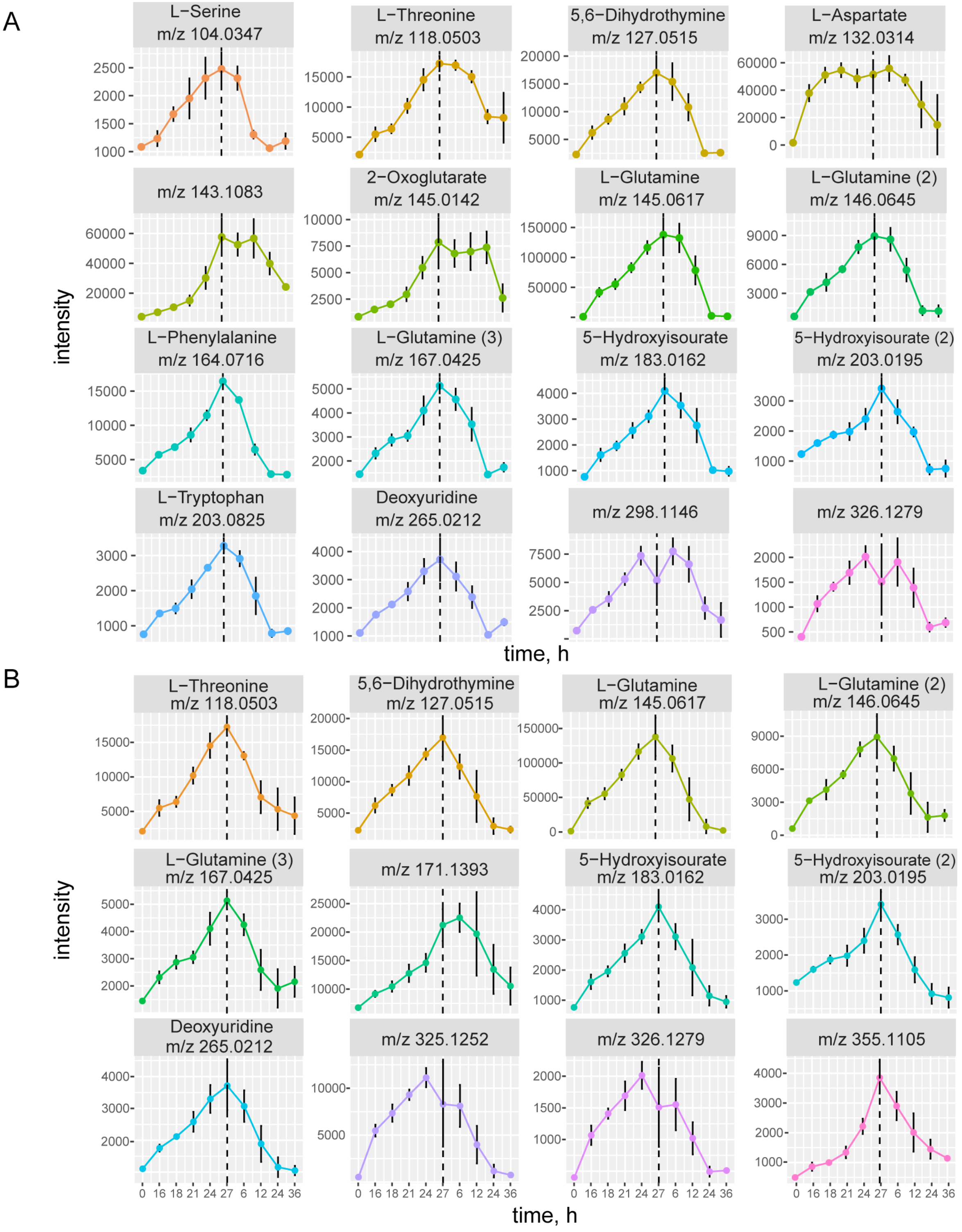

Figure S6. Related to Figure 3. Metabolites produced by *S. cerevisiae* and consumed by LAB (untargeted metabolomics) in presence of rapamycin. (A and B) Metabolites consumed by *L. plantarum* and *L. lactis,* respectively. Metabolite dynamics in yeast and LAB cultures are separated by dashed line. Shown are ions with at

least two-fold change in both accumulation and decrease. Error bars, mean ± s.d., (n = 3 independent biological replicates) in all graphs.

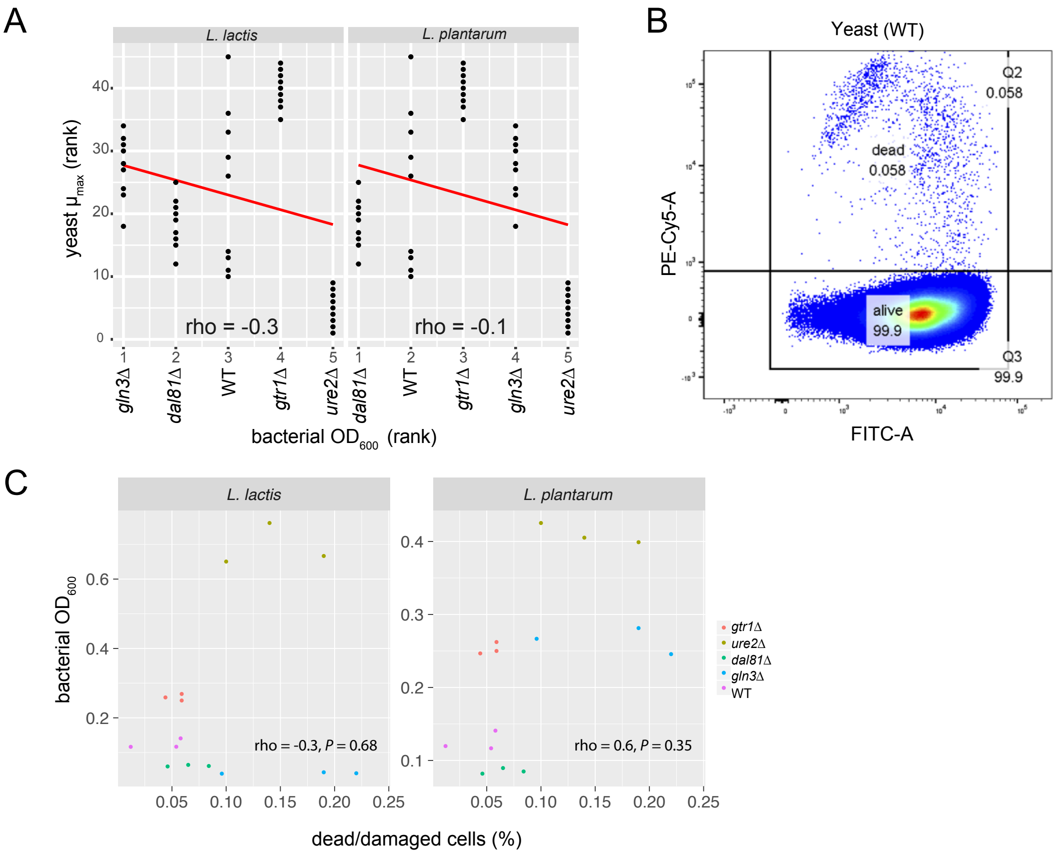

Figure S7. Related to Figure 5. Growth rates and cell death estimates for yeast strains. (A) Growth rate of yeast strains (*ure2Δ, gtr1Δ*, *dal81Δ, gln3Δ* and the wild type) does not correlate with their effect on LAB. (B) Estimation of dead cells using FACS. Shown is the example of raw data for WT cells. Separation of events of live cells (SYTO9 recorded in FITC channel) from dead/membrane-compromised (PI recorded in Cy5 channel). (C) Cell death/damage rate of yeast strains (*ure2Δ, gtr1Δ*, *dal81Δ, gln3Δ* and the wild type) does not correlate with their effect on LAB growth. Dots represent pooled technical replicates from three biological replicates.

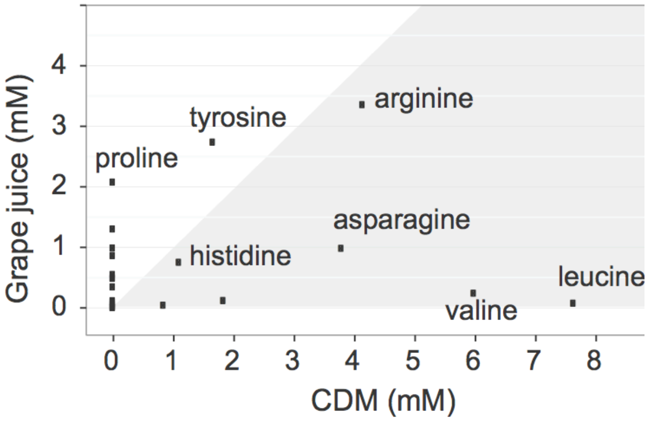

Figure S8. Related to Figure 5. Amino acid content in grape juice as compared with CDM35 medium.

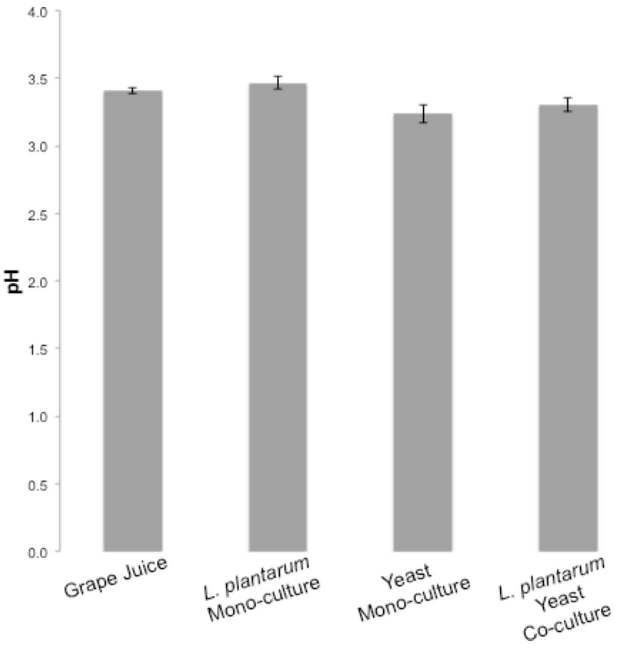

Figure S9. Related to Figure 5. Yeast-LAB co-culture does not change grape juice pH after 24 h. The cultures were treated as is described in Supplemental Experimental Procedures. Error bars, mean ± s.d., (n = 3 independent biological replicates).

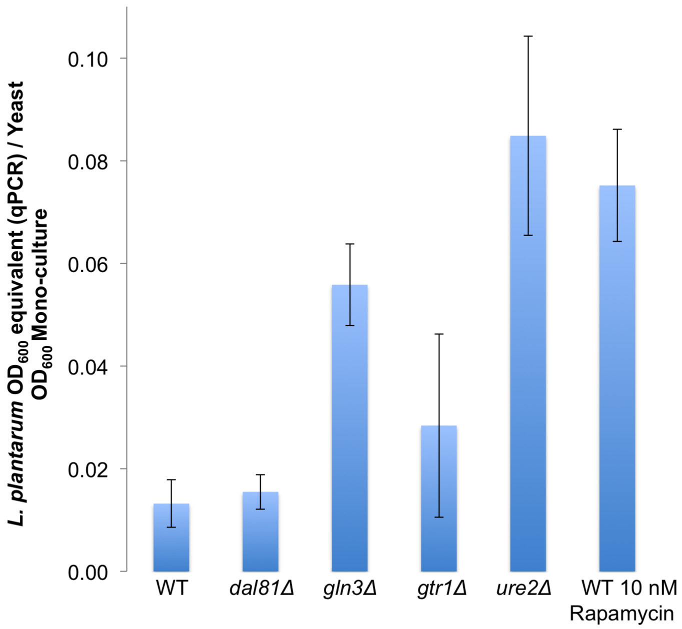

Figure S10. Related to Figure 5. *L. plantarum* growth in grape juice in co-culture with selected *S. cerevisiae* knockout mutants and WT treated with rapamycin. Error bars, mean ± s.d. (n = 3 independent biological replicates).

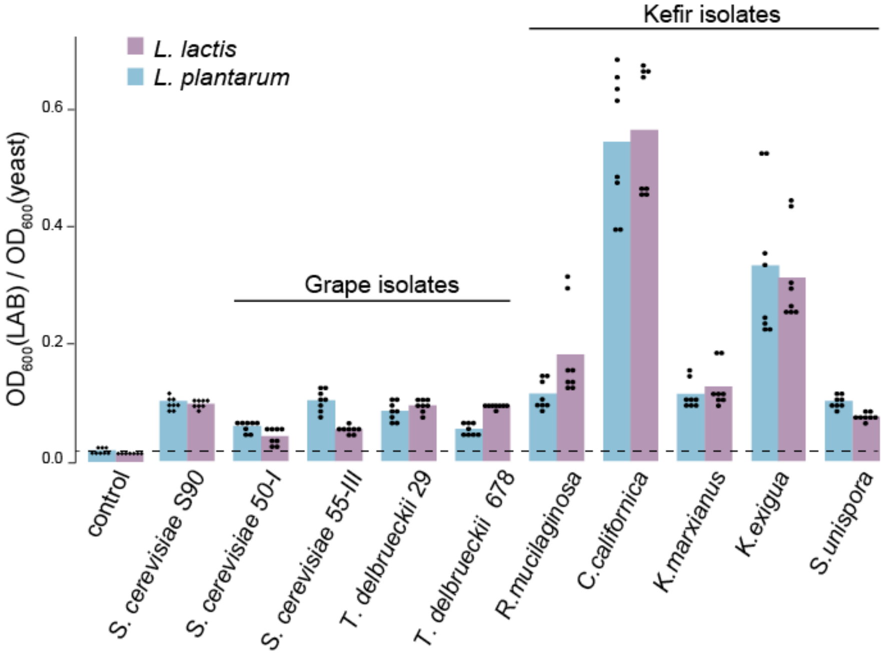

Figure S11. Related to STAR Methods. Effect of CDM35 conditioned medium of different natural yeast isolates on the growth of lactic acid bacteria. Pooled technical replicates from three biological replicates. Dashed line indicates bacterial growth in unconditioned CDM35.

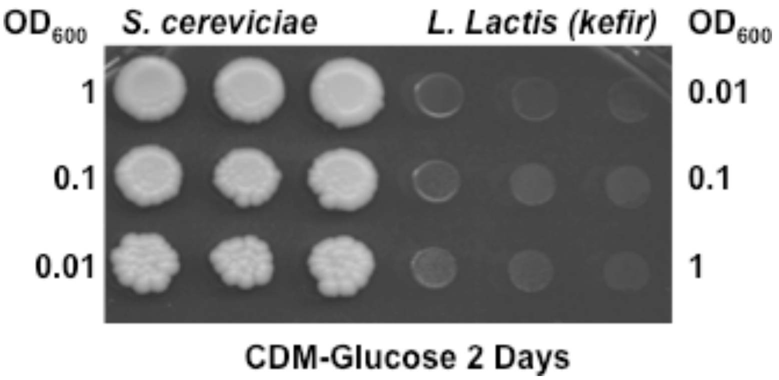

Figure S12. Related to Figure 5E. Unidirectional interaction between yeast and wild *L. lactis* (kefir) in CDM35 (glucose, 2%). OD_{600} values refer to the seed cultures. *S. cerevisiae* growth is independent of the *L. lactis* (kefir) while the *L. lactis* (kefir) grows in proximity to the yeast colonies. This unidirectional interaction becomes mutualistic when glucose is substituted by lactose (Figure 5E).

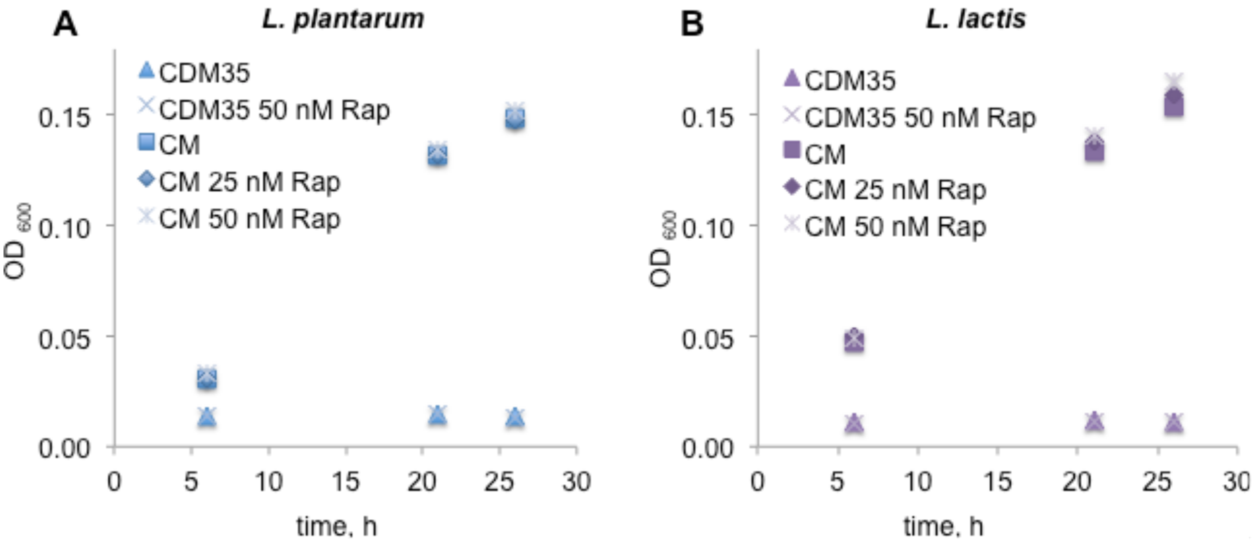

Figure S13. Related to Figure 3. Rapamycin does not affect bacterial growth. Shown is the LAB growth in CDM35 and yeast condition media (CM) with (or without) rapamycin at the indicated concentrations. (A) *L. plantarum*. (B) *L. lactis*. Error bars, mean ± s.d., (n = 3 independent biological replicates).

Supplemental References

Aller, K., Adamberg, K., Timarova, V., Seiman, A., Festsenko, D., and Vilu, R. (2014). Nutritional requirements and media development for Lactococcus lactis IL1403. Appl Microbiol Biotechnol *98*, 5871-5881.

Cocaign-Bousquet, M., Garrigues, C., Novak, L., Lindley, N.D., and Loublere, P. (1995). Rational development of a simple synthetic medium for the sustained growth of Lactococcus lactis. Journal of Applied Bacteriology *79*, 108-116.

Van Niel, E., and Hahn-Hägerdal, B. (1999). Nutrient requirements of lactococci in defined growth media. Applied Microbiology and Biotechnology *52*, 617-627.

Wegkamp, A., Teusink, B., de Vos, W.M., and Smid, E.J. (2010). Development of a minimal growth medium for Lactobacillus plantarum. Letters in applied microbiology *50*, 57-64.

Winzeler, E.A., Shoemaker, D.D., Astromoff, A., Liang, H., Anderson, K., Andre, B., Bangham, R., Benito, R., Boeke, J.D., and Bussey, H. (1999). Functional characterization of the S. cerevisiae genome by gene deletion and parallel analysis. science *285*, 901-906.

Zhang, G., Mills, D.A., and Block, D.E. (2009). Development of chemically defined media supporting high-cell-density growth of lactococci, enterococci, and streptococci. Applied and environmental microbiology *75*, 1080-1087.
